# A neutrophil extracellular trap-related risk score predicts prognosis and characterizes the tumor microenvironment in multiple myeloma

**DOI:** 10.1038/s41598-024-52922-7

**Published:** 2024-01-27

**Authors:** Zhijia Zhao, Yuan Huo, Yufeng Du, Yanan Huang, Hongchen Liu, Chengtao Zhang, Jinsong Yan

**Affiliations:** 1 Liaoning Key Laboratory of Hematopoietic Stem Cell Transplantation and Translational Medicine, Department of Hematology, Liaoning Medical Center for Hematopoietic Stem Cell Transplantation, The Second Hospital of Dalian Medical University, Dalian, 116023 China; 2 Diamond Bay Institute of Hematology, The Second Hospital of Dalian Medical University, Dalian, 116031 China; 3 Blood Stem Cell Transplantation Institute of Dalian Medical University, Dalian, 116023 China; 4 Pediatric Oncology and Hematology Center, The Second Hospital of Dalian Medical University, Dalian, 116023 China

**Keywords:** Cancer, Haematological cancer, Tumour biomarkers

## Abstract

Multiple myeloma (MM) is a distinguished hematologic malignancy, with existing studies elucidating its interaction with neutrophil extracellular traps (NETs), which may potentially facilitate tumor growth. However, systematic investigations into the role of NETs in MM remain limited. Utilizing the single-cell dataset GSE223060, we discerned active NET cell subgroups, namely neutrophils, monocytes, and macrophages. A transcriptional trajectory was subsequently constructed to comprehend the progression of MM. Following this, an analysis of cellular communication in MM was conducted with a particular emphasis on neutrophils, revealing an augmentation in interactions albeit with diminished strength, alongside abnormal communication links between neutrophils and NK cells within MM samples. Through the intersection of differentially expressed genes (DEGs) between NET active/inactive cells and MM versus healthy samples, a total of 316 genes were identified. This led to the development of a 13-gene risk model for prognostic prediction based on overall survival, utilizing transcriptomics dataset GSE136337. The high-risk group manifested altered immune infiltration and heightened sensitivity to chemotherapy. A constructed nomogram for predicting survival probabilities demonstrated encouraging AUCs for 1, 3, and 5-year survival predictions. Collectively, our findings unveil a novel NET-related prognostic signature for MM, thereby providing a potential avenue for therapeutic exploration.

## Introduction

Multiple myeloma (MM) is the second most prevalent hematologic malignancy derived from plasma cells. Constituting 1–2% of all cancers, MM affects an estimated 34,920 individuals in the US and approximately 588,161 globally each year^1^. It is typified by an augmented count of plasma cells in the bone marrow (BM) and increased concentrations of monoclonal immunoglobulins (M-protein) in the serum. These changes lead to complications such as destructive bone lesions, renal impairment, anemia, and hypercalcemia. Notably, the initial symptoms of MM can be ambiguous and resemble other diseases, leading to potential diagnostic and therapeutic delays. For instance, many MM patients experience insidious disease progression with only mildly elevated M-protein concentrations and minimal bone changes, which is easy to overlook. Data from the Surveillance, Epidemiology, and End Results (SEER) program between 2010 and 2016 indicated a 5-year relative survival rate for MM of 53.9%^2^.

To date, there have been different methods for MM staging and grading. The Durie-Salmon staging system, serving as one of the pioneering methods for MM staging, can effectively gauge the tumor burden but presents a certain limitation in the prognostic evaluation^3^. Subsequently, the International Staging System (ISS) was introduced for the preliminary risk stratification of MM. However, ISS lacks adequate considerations about cytogenetics, which play a crucial role in determining the disease's aggressiveness and response to therapy^4^. On the basis of the ISS, the Revised-ISS takes the cytogenetics and lactic dehydrogenase (LDH) levels into consideration^5^. Nonetheless, it addressed only a limited number of cytogenetic abnormalities with high reproducibility, neglecting the prognostic implications of several core genetic abnormalities and their associated phenotypes in the MM microenvironment. Hence, investigation into novel potential biomarkers is essential and meaningful for the prognostic improvement and therapeutic guidance of MM patients.

The BM tumor microenvironment (TME) is pivotal in the pathogenesis and progression of MM. The intricate interaction between MM cells and the BM microenvironment underpins MM cell survival, proliferation, and drug resistance^6^. Neutrophils, the predominant cell population within the BM, engage in numerous interactions with myeloma cells. Research has suggested that neutrophils secrete an array of growth factors and cytokines, such as VEGF, TGF-β, and IL-6 to stimulate the growth and proliferation of myeloma cells^7^. Meanwhile, different enzymes altering the matrix composition can be released by neutrophils to enhance tumor cell migration^8^. Additionally, neutrophils foster angiogenesis^9^, facilitating nutrient supply for MM. In turn, MM cells cultivate a profoundly immunosuppressive BM microenvironment, within which several components including myeloid-derived suppressor cells (MDSCs) and N2 neutrophils are amplified^10^.

Primarily found to neutralize harmful microorganisms, neutrophil extracellular traps (NETs) are extruded from dying neutrophils and present as web-like structures consisting of decondensed DNA chromatin scaffolds and assembled cytosolic and granule proteins. This decondensation of DNA chromatin occurs via citrullination, after which it is expelled from the cell in conjunction with citrullinated histones and neutrophilic cytoplasmic contents rich in granular enzymes–a process termed 'NETosis'^11^. NETs have been reported to present effects of a double-sided nature depending on the immune status and interaction with the TME^12^. From an antitumor immunity standpoint, NETs impede tumor growth by stimulating the immune system: they facilitate neutrophil interactions with T cells, thereby lowering the activation threshold and directly activating T cells. In contrast, NETs can offer a microenvironment suitable for the delivery of protumorigenic proteins to tumor cells^13^. Meanwhile, several studies have indicated that NETs promote the growth and development of tumors via the enhancement of mitochondrial function and induce the activation of corresponding signaling pathways^12,13^.

In hematological neoplasms, there are extremely intricate interactions between tumor cells and the immune system, making the formation of NETs a more universal and complex phenomenon. The importance of NETs has been described in a number of hematological malignancies, including their impact on various aspects of tumorigenesis^14,15^, progression^16^, susceptibility to and severity of infection^17^, and thrombosis^18^. The immunomodulatory role of NETs in leukemia is also likely to be positive, with significant reductions in NETs found during childhood acute lymphoblastic leukemia (ALL) treatment, and increased NETs release as they recover from the disease^19^. Immature granulocytes usually persist in the blood of patients during treatment for hematological malignancies and do not release chromatin for use by NETs after activation^20^, which may partially responsible for the immunodeficiency. The ability of different hematologic tumors to form NETs appears to vary^15,19^, as does their impact on disease pathogenesis.

The significance of NETs in MM is not fully understood. It is known that myeloma cells can induce citrullination of histone H3 and prompt NET formation in neutrophils. Elevated concentrations of NETs or their components have been documented in MM patients and may correlate with disease severity and progression^21^. Citrullination is of great necessity for DNA chromatin decondensation which is one step of NET formation. In the process of citrullination, PAD4 is a key enzyme. One study showed that MM mice treated with BMS-P5, a specific PAD4 inhibitor, presented a noticeable delay in symptom onset and disease progression^22^. This underscores the potential significance of NETs in the pathogenesis and development of MM, suggesting that they could serve as a viable prognostic marker and therapeutic target for the disease^23^.

The advent of single-cell RNA-sequencing (scRNA-seq) technology, coupled with advances in data analysis techniques, offers an unparalleled window into the molecular features of diverse immune cell populations within the TME^24^. Prior research suggests that harnessing gene expression signatures, grounded in the molecular attributes of immune cells extracted from scRNA-seq data, may robustly forecast the prognosis and immunotherapeutic responses of cancer patients^25,26^. In the process of literature retrieval, there is not only no score model based on NET-related genes for MM prognostic evaluation but also no TME assessment and therapeutic guidance on the basis of these genes. Our study examined both single-cell and bulk RNA sequencing data from myeloma samples to pinpoint NET activity associated genes in MM. Leveraging a systematic analysis of these genes, we formulated a risk score model aimed at prognostic prediction for MM patients. Meanwhile, our findings further affirmed the model's stability and its effectiveness in predicting patient prognosis and provided a possible potential direction for MM therapy.

## Results

**Figure 1:**
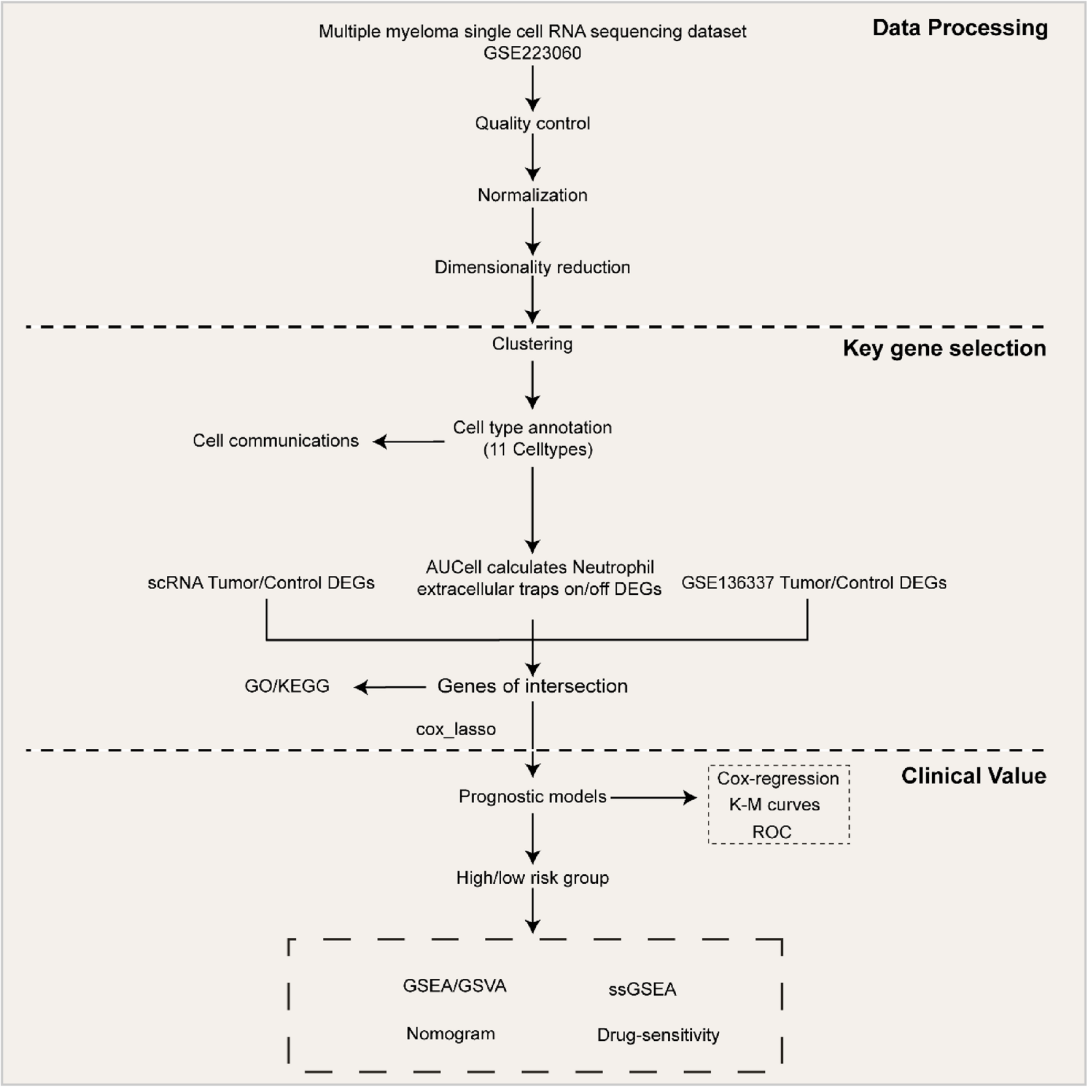
The workflow of the study.

The study's flow chart is illustrated in Fig. 1.

### Single-cell sequencing analysis

**Figure 2:**
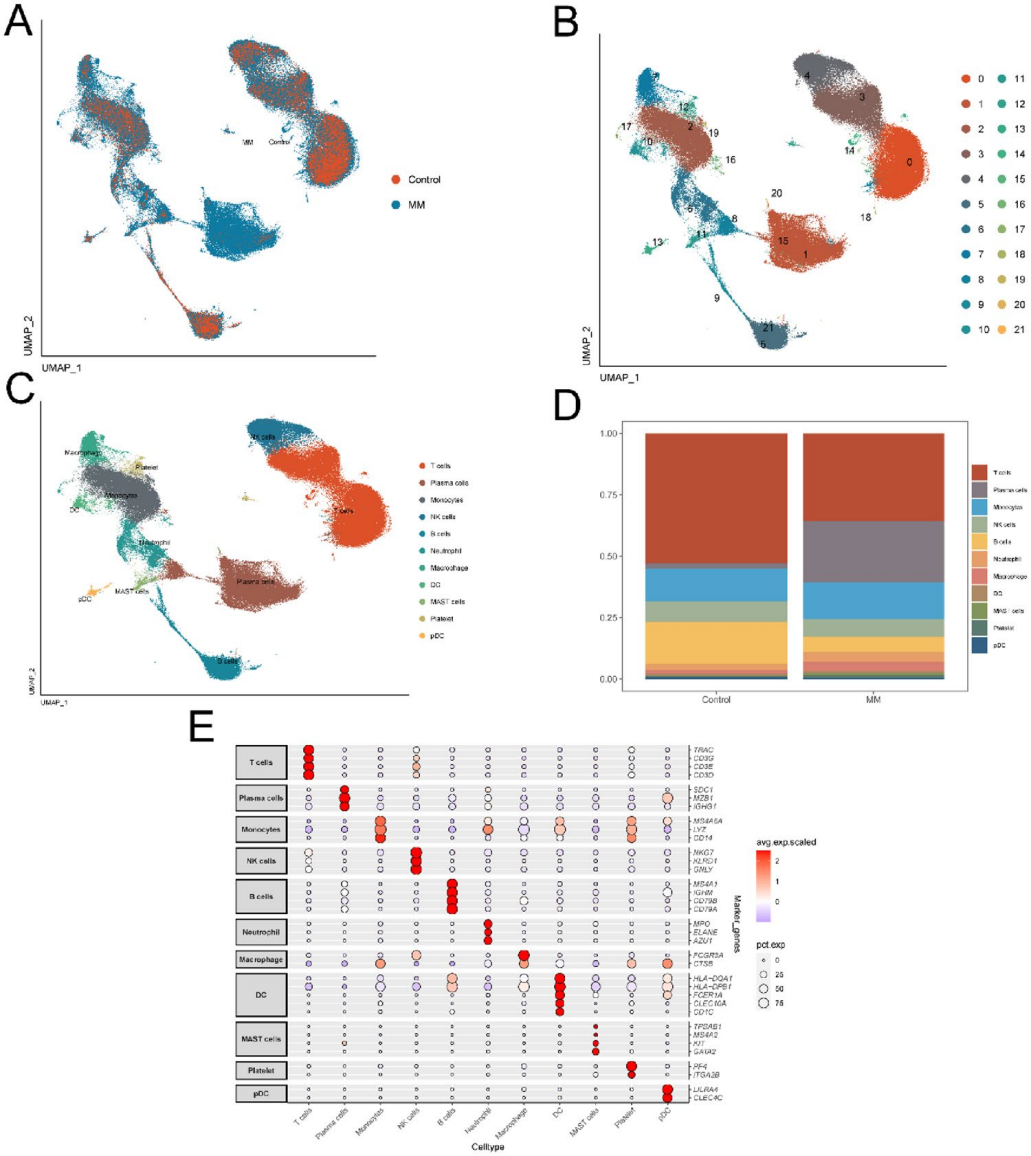
Identification of cell subgroups and expression of marker genes from scRNA-sequencing database. (**A**) UMAP map shows the distribution of MM and control group. (**B**) UMAP map shows the distribution of MM cell subgroups. (**C**) UMAP map shows annotation results of MM cell subgroups. (**D**) Cumulative histogram shows the distribution of cell types in patients with MM and control group. (**E**) Expression profiles of the marker genes in each cell type.

To identify the origins of highly expressed genes, we scrutinized the cell population of MM using the single-cell sequencing dataset GSE223060. After quality control and removal of doublets, we derived single-cell transcriptomes from 166,757 cells. Figure 2A demonstrates that among the 60 samples included, the cell distribution was consistent, suggesting an absence of marked batch effects. This uniformity validates the data for subsequent analyses. Cells were then categorized into 22 distinct clusters, as depicted in Fig. 2B. Each cluster's genetic characteristics enabled us to annotate different cell types using cell type-specific biomarkers. Figure 2C reveals 11 distinct cell types, including T cells, plasma cells, monocytes, NK cells, B cells, neutrophils, macrophages, dendritic cells (DCs), MAST cells, platelets, and plasmacytoid dendritic cells (pDCs). The dot plots in Fig. 2E illustrate specific genes for each cell type, while Fig. 2D displays the proportions of these cell types across samples.

### Identification of neutrophil extracellular trap active cells

**Figure 3:**
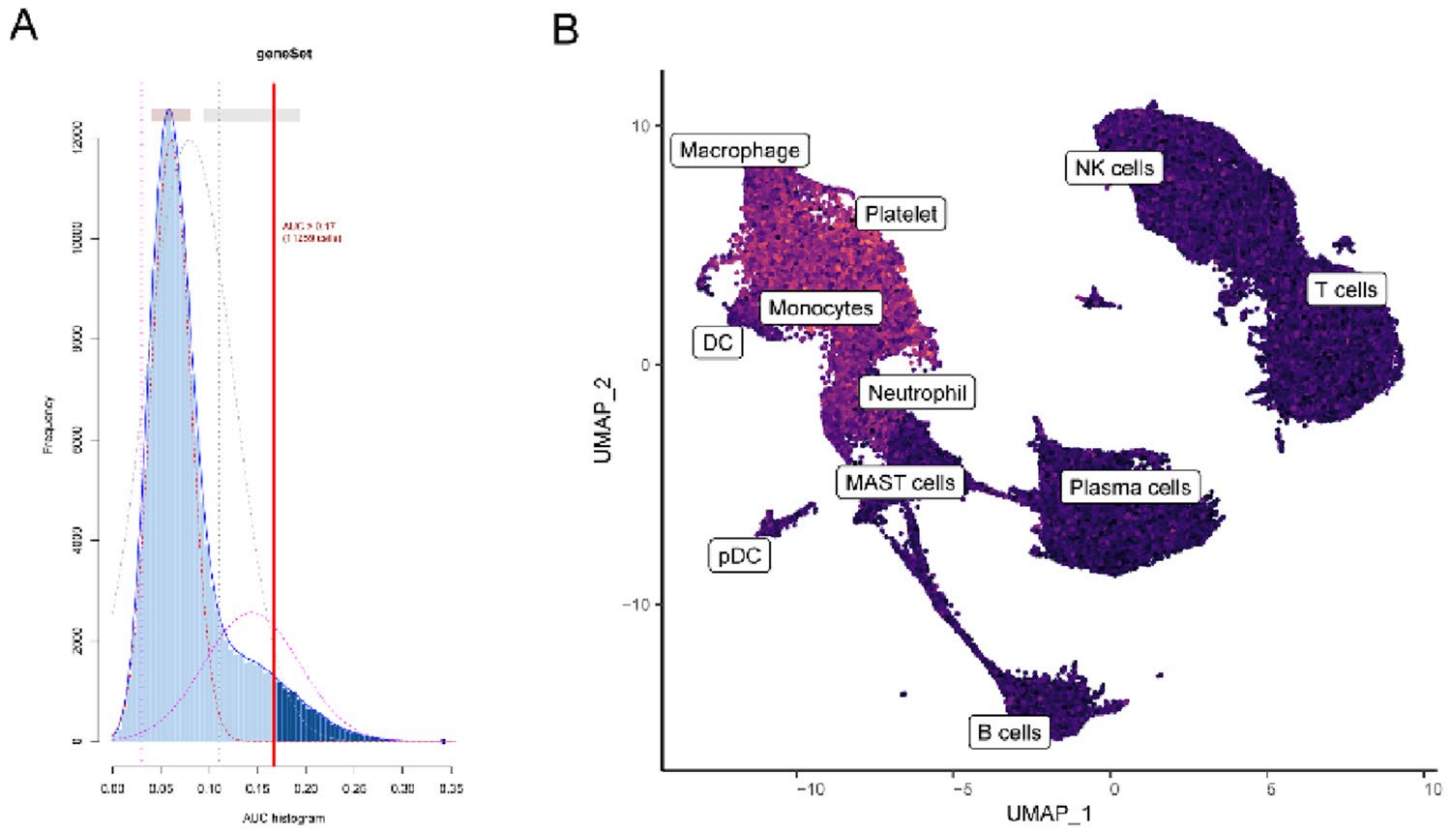
Identification of active cell subgroups. (**A**) AUC score of the NET-related marker genes, the threshold value was 0.17. (**B**) UMAP colorogram shows the score of cell activity. The brighter the color, the higher the activity.

We examined the expression patterns of NET-related genes at the single-cell level within active cell subgroups. Utilizing the optimal threshold to ascertain cell activity, we identified 11,259 cells exhibiting NET activity. Cell clusters with AUC values exceeding 0.17 displayed high NET activity, whereas those with AUC values below 0.17 exhibited low NET activity, as depicted in Fig. 3A. Figure 3B presents the UMAP diagram of these active cells, indicating that neutrophils, monocytes, and macrophages were predominantly active.

### Pseudo-time trajectories analysis

**Figure 4:**
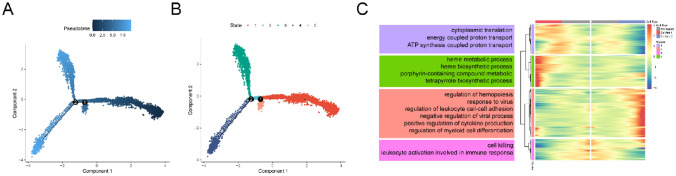
Transcriptional trajectory analysis revealed transcriptional patterns in NET activated subgroups. (**A**) The pseudo-time color gradient transitions from dark to light blue. (**B**) The pseudo-time trajectory is divided into three different states by Monocle 2. (**C**) The DEGs of different branches (different cell fates) shown in heatmap. The top GO BP pathways of different clusters in heatmap were listed nearby.

Utilizing the definitive NET-activated subgroups, we constructed a transcriptional trajectory to identify key gene expression programs governing MM progression. The trajectory's transcriptional states highlighted distinct paths, as depicted in Fig. 4A and B.

To decipher the molecular underpinnings of this transformation, we investigated the genes influencing MM cell fate. Genes predominantly expressed in the pre-branch were chiefly associated with 'cell killing' and 'leukocyte activation involved in immune response' GO BP pathways. Meanwhile, genes related to 'regulation of hemopoiesis', 'response to virus', and 'regulation of leukocyte cell–cell adhesion' were predominant in cell fate 2. Conversely, cell fate 1 exhibited high expression of genes linked to 'cytoplasmic translation', 'energy-coupled proton transport', and 'ATP synthesis coupled proton transport', as illustrated in Fig. 4C and Table S1.

### Cellular communication patterns in the MM microenvironment

**Figure 5:**
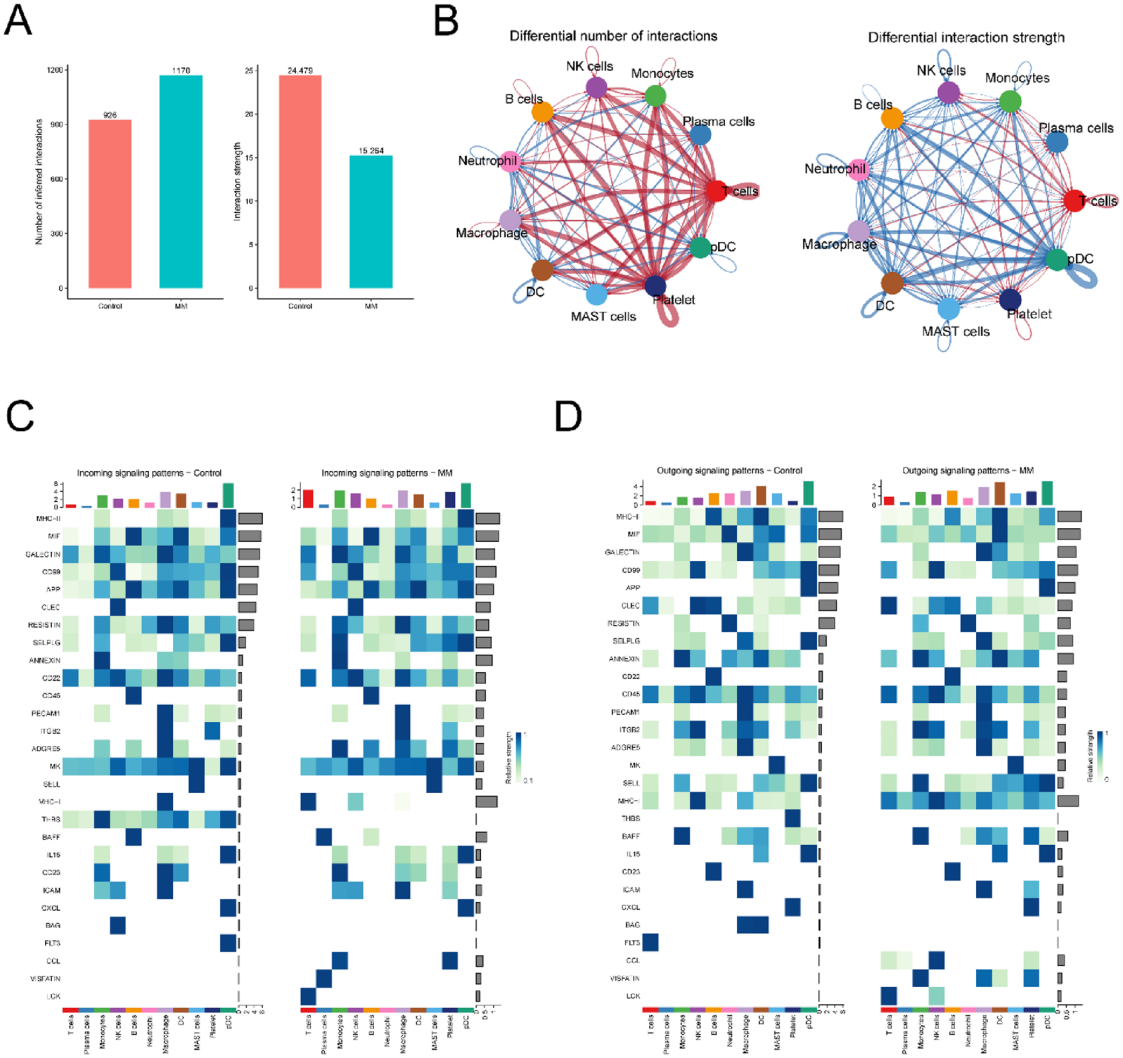
Overall pattern of intercellular communication analysis. (**A**) Bar plot shows the interaction number and strength between MM and normal. (**B**) The network diagram displays the number and strength of interactions between cell types in the MM and control groups. The red bands represent an increase or enhancement in the number and strength of interactions, while the blue bands represent a decrease or weakening in the number and strength of interactions. (**C**) Heatmap depicting signals contributing the most to the outgoing signaling pathways in MM and normal. (**D**) Heatmap depicting signals contributing the most to the incoming signaling pathways in MM and normal.

To delve deeper into the cellular interaction network within the MM microenvironment, we employed the 'CellChat' R package to discern variations in cell-to-cell communication between the MM and control groups. Comparison to normal tissues revealed an increase in the quantity of interactions among MM samples, accompanied by a decrease in the intensity of these interactions, as illustrated in Fig. 5A. Furthermore, Fig. 5B illustrates that, in the case of the majority of cell interactions, both the quantity and strength of these interactions exhibited an augmentation when contrasted with normal tissues. These findings underscore the intricate nature of the microenvironment. The outgoing and incoming signaling patterns for both normal and MM tissues are distinctly illustrated in Fig. 5. For instance, the macrophage migration inhibitory factor (MIF) signal targeting DCs was diminished in MM (Fig. 5C), and the lymphocyte specific protein tyrosine kinase (LCK) signal originating from T cells was reduced in MM (Fig. 5D).

**Figure 6:**
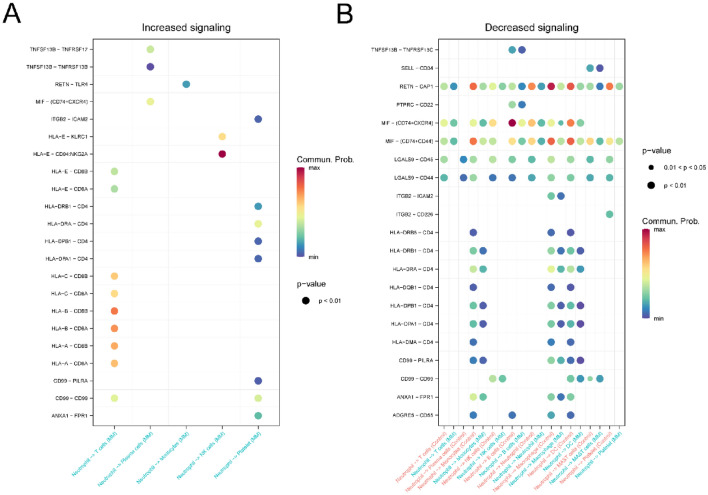
Comparison significant ligand-receptor pairs between neutrophil and other cells. (**A**) Significantly increased ligand receptor pairs in the MM group. (**B**) Significantly reduced ligand receptor pairs in the MM group.

Additionally, we examined receptor ligands potentially mediating communication between neutrophils and other immune cells. Notably, neutrophils communicated with NK cells through the HLA-E-KLRC1 and HLA-E-CD94:NKG2A pathways, which were absent in normal tissues. This implies a role for neutrophil-derived HLA-E in MM progression, as depicted in Fig. 6A and B.

**Figure 7:**
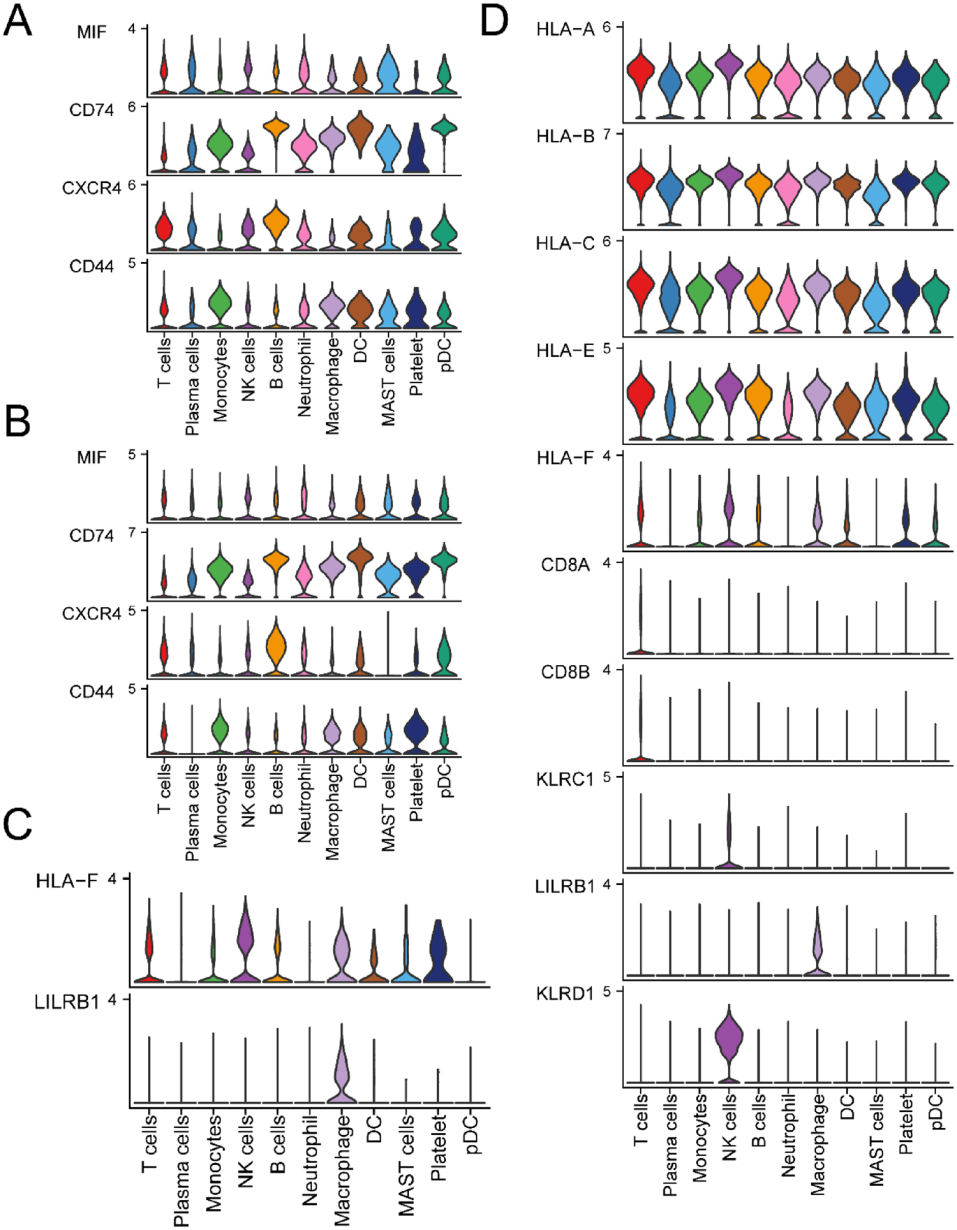
The expression of MIF and MHC-I signaling pathways as ligand receptors in tissues. (**A**) The expression distribution of MIF signaling ligand receptors in the control group. (**B**) The expression distribution of MIF signaling ligand receptors in the MM group. (**C**) The expression distribution of MHC-I signaling ligand receptors in the control group. (**D**) The expression distribution of MHC-I signaling ligand receptors in the MM group.

Subsequently, we delved into the expression of MIF and MHC-I pathway genes across different cells in both normal and MM tissues. In comparison to control tissues, the expression of the MIF ligand in neutrophils was notably reduced in MM, and the expression of its receptors in NK cells was also decreased (Fig. 7A and B). In contrast, the expression of the MHC-I ligand in neutrophils remained unchanged in MM, and a similar stability was observed in the expression of its receptors in NK cells (Fig. 7C and D). This provides insight into the diminished communication intensity of the MIF pathway between neutrophils and NK cells in MM tissues.

### Enrichment analysis of differentially expressed genes related to neutrophil extracellular traps in MM

**Figure 8:**
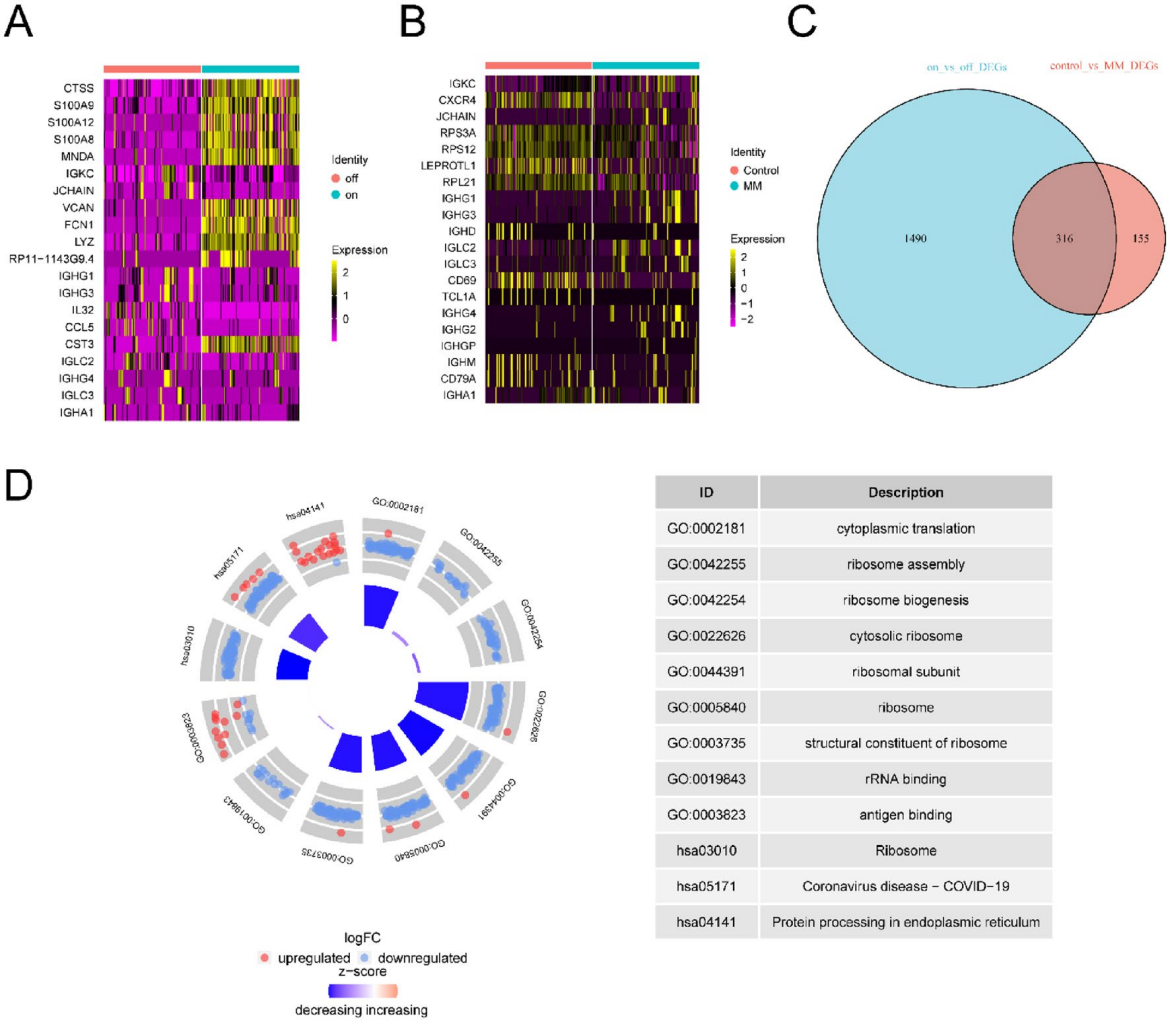
Enrichment analysis of DEGs related to NET activity in MM. (**A**) The heatmap shows the significantly DEGs in NET active cells of MM. (**B**) The heatmap shows the significantly DEGs between MM and controls in single-cell dataset. (**C**) The Venn diagram highlights the key genes. (**D**) The circle diagram shows GO and KEGG enrichment results of intersection genes.

A total of 1,806 DEGs were discerned between NET active and inactive cells (Table S2), exhibiting significant differences (adjusted *p* value < 0.05; | Log2-fold change |> 0.25). The heatmap in Fig. 8A displays the top 10 upregulated (CTSS, S100A9, S100A12, S100A8, MNDA, VCAN, FCN1, LYZ, RP11-1143G9.4, CST3) and downregulated genes (IGHG3, IGKC, IGHG1, JCHAIN, IGHA1, IGLC3, IGLC2, IGHG4, IL32, CCL5).

Differential expression analysis between MM samples and healthy controls was conducted separately for single-cell and bulk transcriptome datasets. From the single-cell dataset, 471 significant DEGs were identified (adjusted *p* value < 0.05; | Log2-fold change |> 0.25), as depicted in Table S3. Figure 8B's heatmap showcases the top 10 upregulated (IGKC, IGHG3, IGLC3, IGHG1, IGHA1, JCHAIN, IGLC2, IGHG4, IGHG2, IGHGP) and downregulated genes (CD69, IGHM, TCL1A, CXCR4, IGHD, IGHD, LEPROTL1, RPS3A, CD79A, RPS12).

An overlap between the two DEG sets revealed 316 intersection DEGs, as visualized in Fig. 8C and detailed in Table S4.

To elucidate the biological functions of these intersection DEGs, we undertook enrichment analysis for GO terms and KEGG pathways. GO analysis, detailed in Table S5, indicated enrichment in biological processes such as cytoplasmic translation, ribosome assembly, and ribosome biogenesis. CC were dominated by features such as cytosolic ribosome and ribosomal subunit, while MF featured rRNA binding and antigen binding (Fig. 8D). Prominent KEGG pathways (Table S6) included Coronavirus disease–COVID-19, Ribosome, and Protein processing in the endoplasmic reticulum (Fig. 8D).

### Construction and verification of prognostic risk model

**Figure 9:**
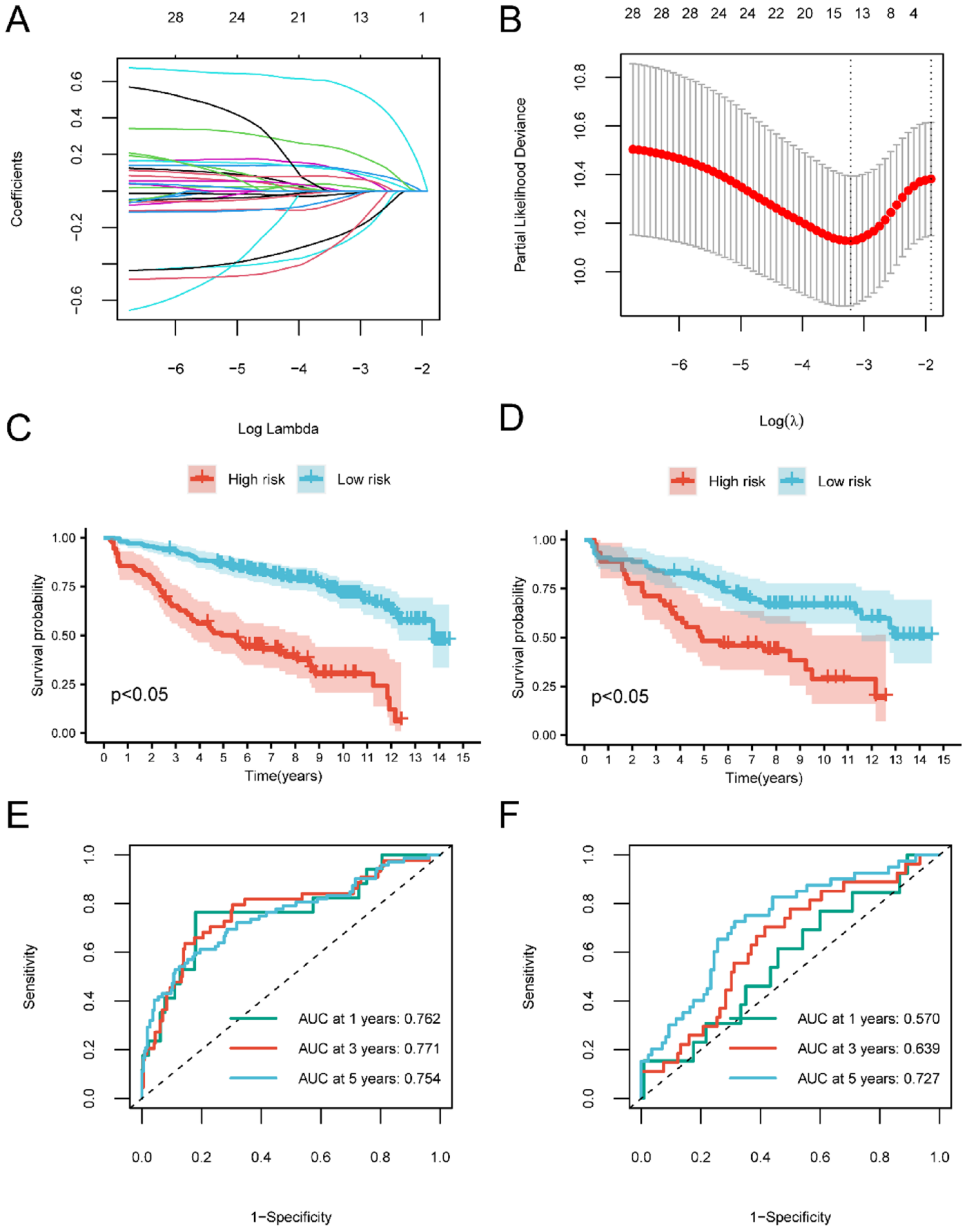
Cox and LASSO regression analysis of the MM dataset. (**A**) Change trajectory of LASSO regression independent variable, the abscissa represents the logarithm of the independent variable λ, and the ordinate represents the coefficient of the independent variable. (**B**) Confidence interval under each lambda in LASSO regression. (**C**) The survival curve of patients in high- and low-risk groups from training cohort, respectively. (**D**) The survival curve of patients in low- and high-risk groups from validation cohort, respectively. Red represents the high-risk group, and blue represents the low-risk group. (**E**) 1-, 3-, and 5-year time-dependent ROC curves of models for training cohorts. (**F**) 1-, 3-, and 5-year time-dependent ROC curves of models for validation cohorts.

Through univariate Cox analysis of the 316 intersection DEGs (between 1806 DEGs form NET active vs. inactive cells and 471 DEGs from MM samples vs. healthy controls), 28 NET-related prognostic genes significantly associated with MM prognosis were identified (*p* < 0.05) (Table S7). Using random sampling, 70% (n = 291) of the MM samples (n = 420) were allocated to the training set, and the remaining 30% (n = 129) formed the validation set. Genes that have no or little effect on the effectiveness of the predictive model are defined as redundant genes. Redundant genes within the training set were pruned via LASSO regression analysis, with the seed parameter set at 44. This yielded 13 NET-related hub genes significantly tied to MM patient prognosis (Table S8), as illustrated in Fig. 9A and B, which were utilized to construct prognostic models. The other 15 genes out of the 28 genes were used as redundant genes and excluded from the model.

To validate the models crafted from these 13 gene signatures, samples were categorized into low- and high-risk cohorts, using the median risk value as a delimiter. Kaplan–Meier survival curves were plotted for both the training (Fig. 9C) and validation cohorts (Fig. 9D). Evidently, patients in the high-risk cohort exhibited notably poorer prognoses than their low-risk counterparts across both datasets. To gauge the predictive capacity of the model, ROC curves were generated (Fig. 9E and F). In the training set, the 1-, 3-, and 5-year survival AUC values were 0.762, 0.771, and 0.754, respectively (Fig. 9E). Correspondingly, in the validation set, these AUC values were 0.570, 0.639, and 0.727 (Fig. 9F).

### GSEA and GSVA

**Figure 10:**
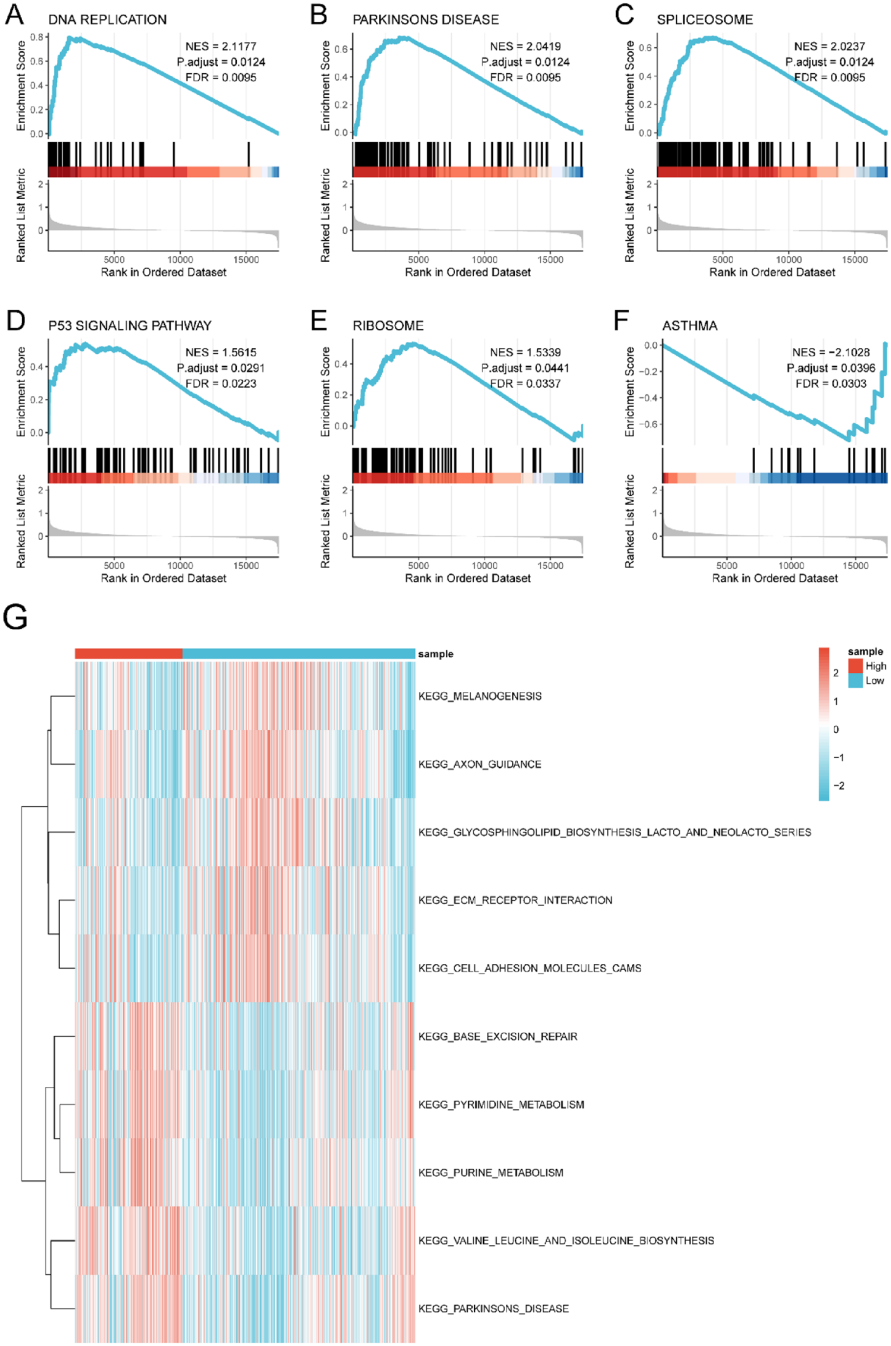
GSEA and GSVA of significantly enriched pathways. DNA REPLICATION (**A**), PARKINSONS DISEASE (**B**), SPLICEOSOME (**C**), P53 SIGNALING PATHWAY (**D**), RIBOSOME (**E**), ASTHMA (**F**). (**G**) GSVA of significantly enriched pathways.

To elucidate the potential mechanisms of the DEGs, we employed GSEA. Utilizing the MSigDB Collection, we identified the most significantly enriched signaling pathways according to their normalized enrichment score (NES) (Table S9). Significantly enriched pathways in MM included DNA REPLICATION (NES = 2.1177, adjusted *p* = 0.0124, FDR = 0.0095, Fig. 10A), PARKINSON'S DISEASE (NES = 2.0419, adjusted *p* = 0.0124, FDR = 0.0095, Fig. 10B), SPLICEOSOME (NES = 2.0237, adjusted *p* = 0.0124, FDR = 0.0095, Fig. 10C), P53 SIGNALING PATHWAY (NES = 1.5615, adjusted *p* = 0.0291, FDR = 0.0223, Fig. 10D), RIBOSOME (NES = 1.5339, adjusted *p* = 0.0441, FDR = 0.0337, Fig. 10E), and ASTHMA (NES = -2.1028, adjusted *p* = 0.0396, FDR = 0.0303, Fig. 10F). Additionally, using the MSigDB Collection for GSVA, we highlighted the top 5 pathways with the most significant differential expression between low- and high-risk groups. These findings are visualized in the pathway activity heatmap (Fig. 10G and Table S10).

### Immune infiltration analysis

**Figure 11:**
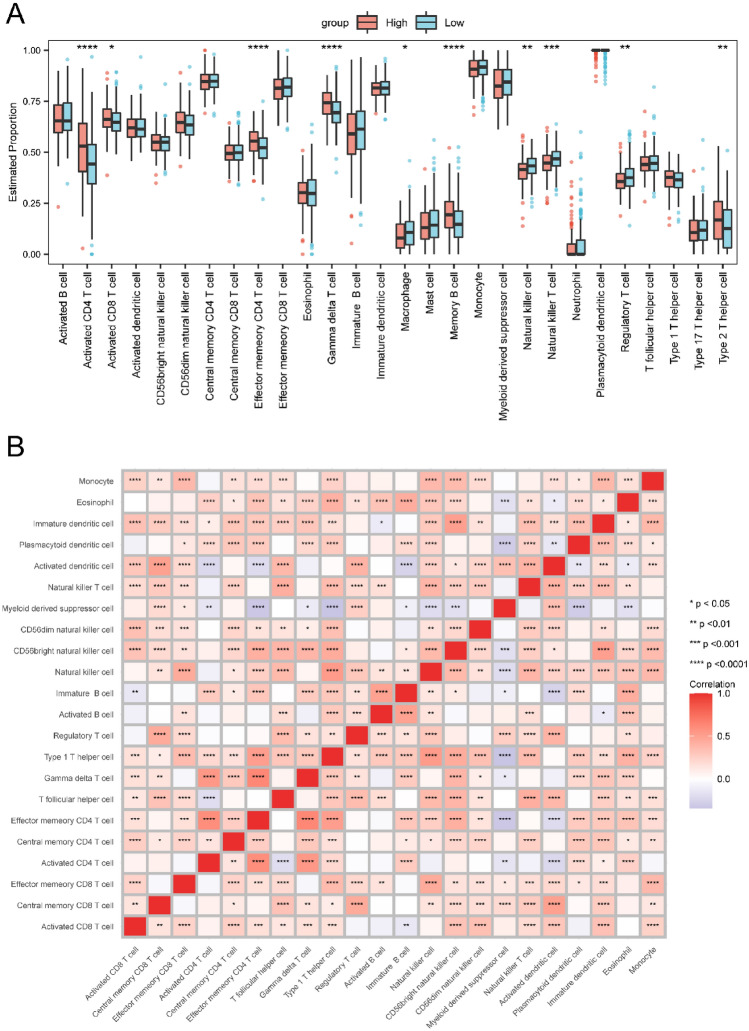
Distinction of immune infiltrations between the high and low risk groups. (**A**) Boxplot shows the estimated proportion of immune cells between low- and high-risk groups. (**B**) Correlation among immune cells. Asterisks represented *p* value (****p < 0.0001, ***p < 0.001, **p < 0.01, *p < 0.05).

We examined the infiltration levels of 28 immune cell types between the low- and high-risk groups using the ssGSEA method. The infiltration levels of different immune cells, including activated CD4^+^ T cells, activated CD8^+^ T cells, effector memory CD4^+^ T cells, gamma delta T cells, macrophages, memory B cells, NK cells, NK T cells, regulatory T cells, and type 2 T helper cells, exhibited significant differences between the two groups (*p* < 0.05, Fig. 11A). While most immune cells exhibited positive correlations with each other, a subset demonstrated negative correlations. Specifically, MDSCs, effector memory CD4^+^ T cells, type 1 T helper cells, memory B cells, CD56bright NK cells, pDCs, and NK cells infiltration levels were negatively correlated (Fig. 11B).

**Figure 12:**
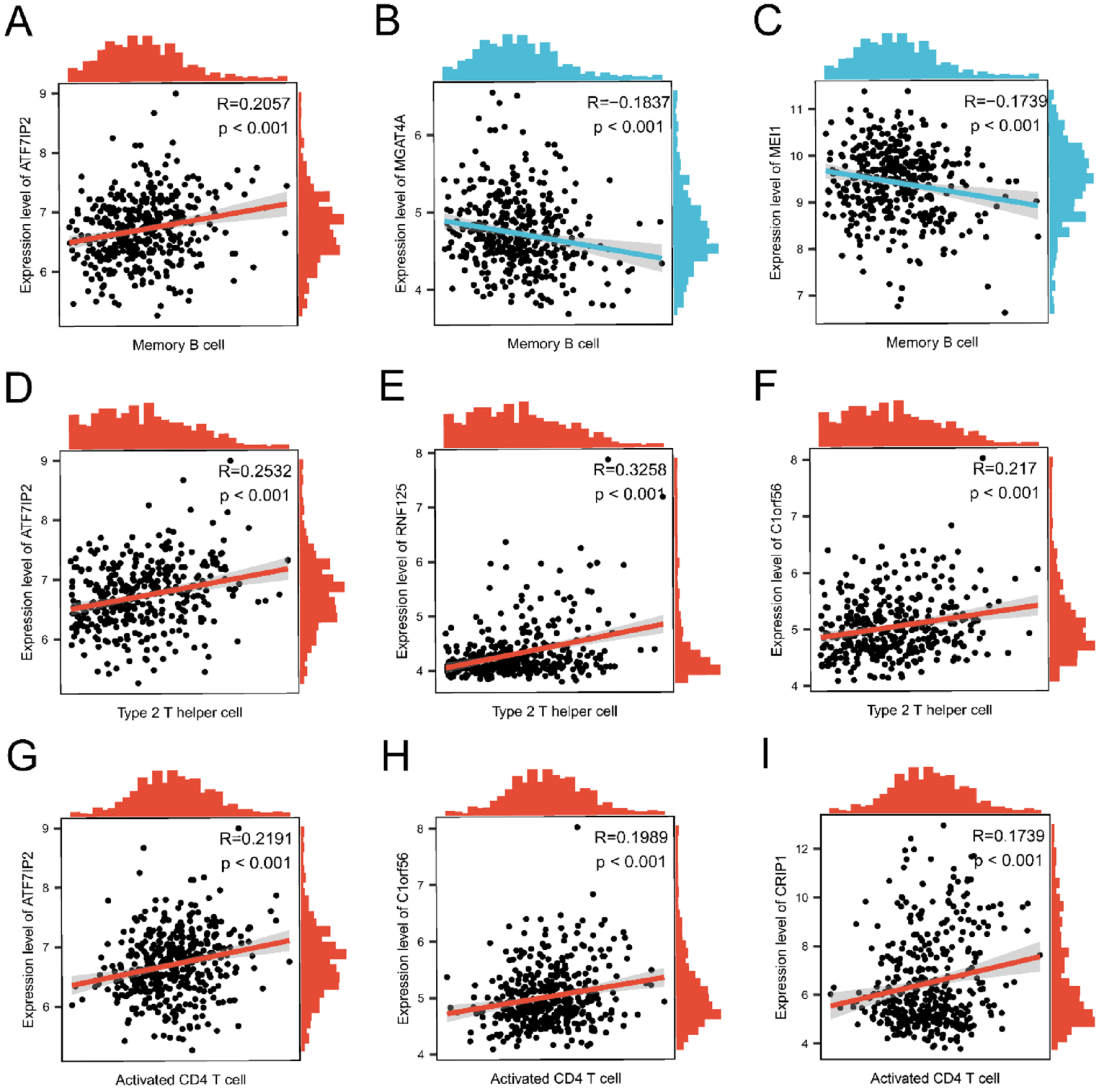
Correlation between immune cells and genes. Correlation of gene ATF7IP2 (**A**), MGAT4A (**B**) and MEl1 (**C**) with Memory B cell; Correlation of gene ATF7IP2 (**D**), RNF125 (**E**) and C1orf56 (**F**) with Type2 T helper cell; Correlation of gene ATF7IP2 (**G**), C1orf56 (**H**) and CPIP1 (**I**) with Activated CD4^+^ T cell.

Additionally, we observed significant correlations between each hub gene and its corresponding immune cells (Fig. 12A–I). Notably, the genes ATF7IP2 (R = 0.2057, *p* < 0.001), MGAT4A (R = -0.1837, *p* < 0.001), and MEl1 (R = -0.1739, *p* < 0.001) were significantly associated with memory B cells (Fig. 12A–C). Genes ATF7IP2 (R = 0.2532, *p* < 0.001), RNF125 (R = 0.3258, *p* < 0.001), and C1orf56 (R = 0.217, *p* < 0.001) had a significant association with type 2 T helper cells (Fig. 12D–F), whereas ATF7IP2 (R = 0.2191,* p* < 0.001), C1orf56 (R = 0.1989, *p* < 0.001), and CPIP1 (R = 0.1739, *p* < 0.001) were significantly related to activated CD4^+^ T cells (Fig. 12G–I).

### Construction and verification of the nomogram

**Figure 13:**
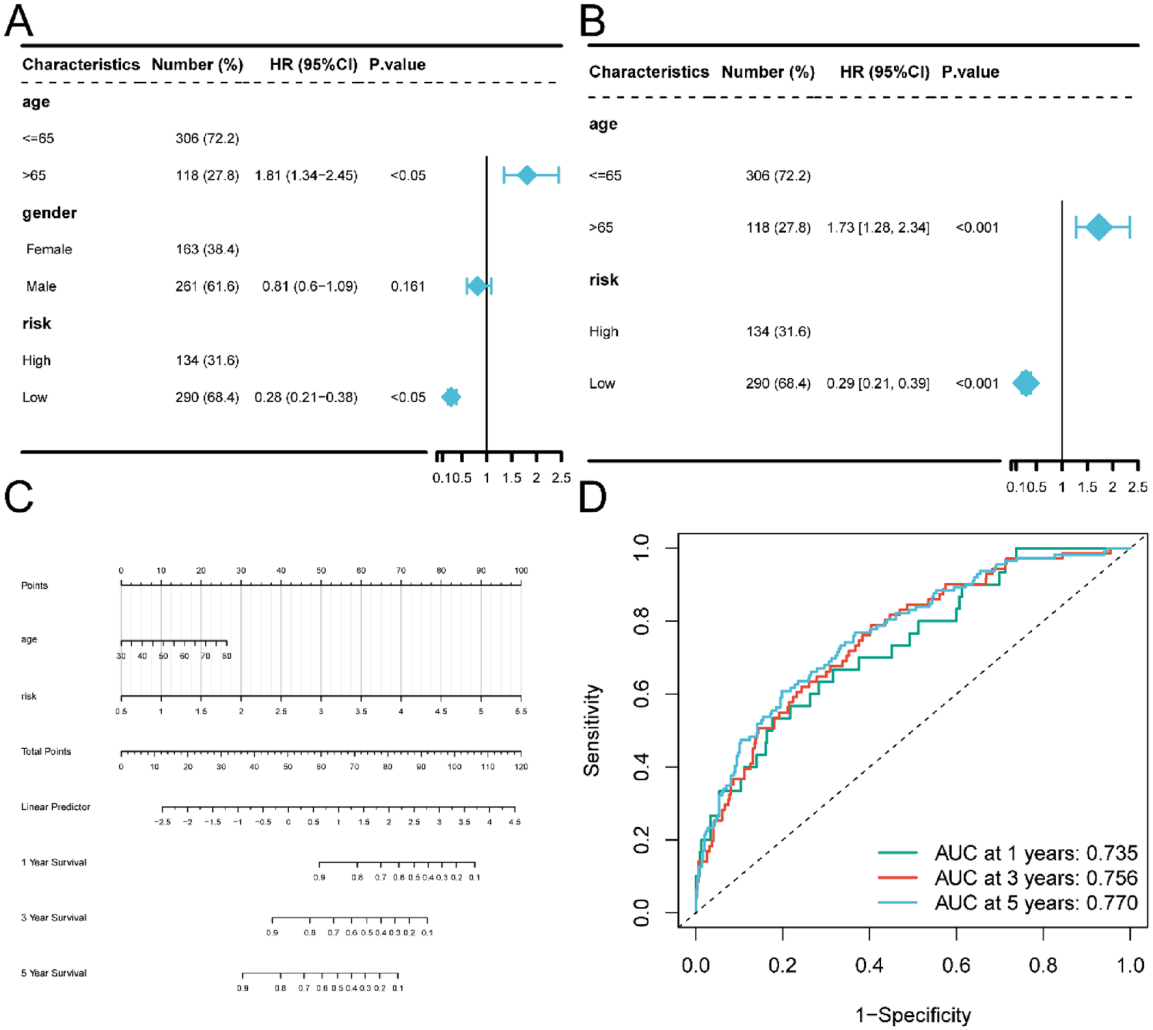
Risk score is an independent prognostic factor for clinical characteristics. (**A**) Forest map shows the results of univariate Cox regression analysis performed on clinical characteristics. (**B**) Forest map shows the results of multivariate Cox regression analysis performed on clinical characteristics. (**C**) The nomogram of the prediction model. The line segment represents the contribution of the clinical factor to the outcome events, total points represent the total score of the sum of the corresponding individual scores of the value of all variables, and the bottom three lines represent the prognosis of 1-, 3-, and 5-year survival corresponding to each value point. (**D**) 1-, 3-, and 5-year time-dependent ROC curves of nomogram.

To ascertain the role of the risk score as an independent prognostic factor, we conducted both univariate and multivariate Cox regression analyses considering clinical characteristics such as age, sex, and risk. Our findings affirm that the risk score stands as an independent prognostic risk factor for patients, irrespective of the Cox regression analysis employed (Fig. 13A and B). Utilizing multivariate Cox regression analysis, we constructed a nomogram, demonstrating that the risk score can significantly forecast clinical outcomes (Fig. 13C). We employed the ROC curve to evaluate the predictive efficacy of the nomogram concerning patient prognosis. The AUC values for 1-, 3-, and 5-year survival were 0.735, 0.756, and 0.770, respectively (Fig. 13D).

### Drug susceptibility analysis

**Figure 14:**
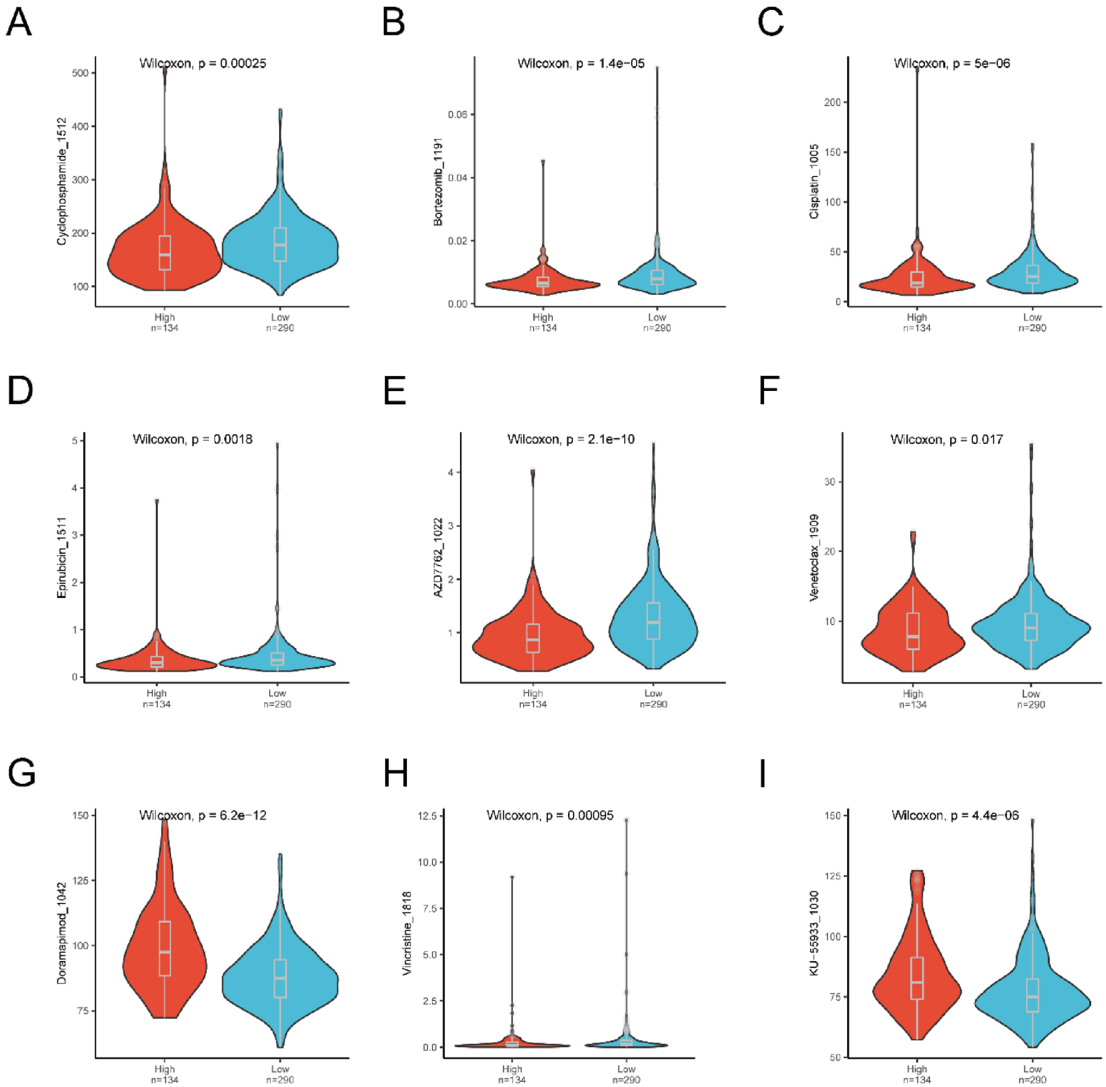
Drug susceptibility between the low- and high-risk groups. (**A**) Difference in sensitivity to Cyclophosphamide_1512 between low- and high-risk groups. (**B**) Difference in drug sensitivity of Bortezomib_1191 between low- and high-risk groups. (**C**) Differences in drug susceptibility to Cisplatin_1005 between low- and high-risk groups. (**D**) Difference in Podophyllotoxin Epirubicin_1511 drug sensitivity between low- and high-risk groups. (**E**) Differences in AZD7762_1022 susceptibility between low- and high-risk groups. (**F**) Differences in drug sensitivity of Venetoclax_1909 between the low- and high-risk groups. (**G**) Differences in susceptibility to Doramapimod_1042 between low- and high-risk groups. (**H**) Difference in Vincristine_1818 susceptibility between low- and high-risk groups. (**I**) Difference in drug sensitivity to KU-55933_1030 between low- and high-risk groups.

We investigated the ability of the risk score to predict chemotherapeutic sensitivity in MM patients. We assessed several agents, namely cyclophosphamide_1512, bortezomib_1191, cisplatin_1005, epirubicin_1511, AZD7762_1022, venetoclax_1909, doramapimod_1042, vincristine_1818, and KU-55933_1030, for their therapeutic effectiveness in MM treatments (Fig. 14, Table S11). Our findings indicate that patients with a high-risk score might have increased sensitivity to conventional chemotherapy drugs, specifically cyclophosphamide_1512, cisplatin_1005, epirubicin_1511, vincristine_1818, and the proteasome inhibitor (PI) bortezomib_1191. This suggests that a combination of PI and multidrug chemotherapy may be optimal for this patient cohort.

## Discussion

With immunotherapy advancing and gaining more attention, an increasing number of biomarkers have been explored for immunotherapy response prediction^27^. Due to the nonnegligible and vital effects of the TME on tumor occurrence and development, influence of the TME on cancer immunotherapeutic efficacy has been extensively examined, and TME-related biomarkers have attracted heightened attention^28^. However, there remains a paucity of reliable biomarkers and risk score models reflecting the roles of the tumorigenic TME in immunotherapeutic responses and prognosis in MM. The evolution of scRNA-seq technology offers a comprehensive lens into the molecular profiles of tumor-infiltrating immune cells within the TME^29^. In this study, 41 MM samples were analyzed via single-cell sequencing, and 11 distinct cell types were identified. We observed a higher proportion of neutrophils in myeloma samples than in normal controls, unlike lymphocytes which were suppressed by elevated tumorigenic plasma cells (Fig. 1D). The ability of neutrophils in MM to form NETs also appears to be like other hematological tumors and does not directly correlate with their count^19^, which needs to be further determined. We utilized NET-related genes sourced from the work of Zhang et al.^30^, for the subsequent evaluation of NET activity via the AUCell algorithm. Cell populations with AUC values exceeding 0.17 were characterized by high NET activity, suggesting that NETs might modulate these cells, influencing tumorigenesis and progression. Subsequently, the pivotal genes governing NET activity were further pinpointed by combining the results of the difference and enrichment analyses.

The 13-gene signature effectively differentiates patients into low- and high-risk subpopulations. Across all training and validation sets, our signature demonstrated great consistency and stability. Specifically: (1) patients from distinct risk subpopulations were clearly distinguishable; (2) high-risk subpopulation patients exhibited a poor prognosis; (3) tumor immune microenvironments between low- and high-risk subpopulations exhibited significant differences; and (4) the signature's diagnostic values for 1-year, 3-year, and 5-year survival rates were commendable. We found that the 1, 3, and 5-year AUC values of our NET-related prognostic model were higher than of the UAMs GEP70^31^ model in the same dataset (GSE136337), which suggested its good prognostic predictive accuracy (Supplementary Fig. 1A, B). Interestingly, we chose to externally validate the NET-related prognostic model with a dataset of screened myeloma cells (GSE4581), which still resulted in a significant difference in prognosis between the differentiated low and high-risk groups (Supplementary Fig. 2A, B).

Among the 13 signature-associated genes (RNF125, NPM1, CRIP1, HIST1H1C, C1orf56, S100A6, GAPDH, CCND1, RHOH, ANKRD28, MEI1, MGAT4A and ATF7IP2), RNF125, NPM1, CRIP1, HIST1H1C, C1orf56, S100A6 and GAPDH were risk genes, while CCND1, RHOH, ANKRD28, MEI1, MGAT4A and ATF7IP2 served as protective genes. Among those risk genes, RNF125 was significantly linked to a high score and poor prognosis. RNF125, an E3 ubiquitin ligase, is involved in tagging specific proteins, leading to their ubiquitination and subsequent degradation. In immune processes, RNF125 ubiquitinates key signaling molecules, influencing their stability and function. This mechanism is essential in MM and is regarded as the target of PIs such as bortezomib^32^. Additionally, RNF125 may function as a positive regulator in the T-cell receptor signaling pathway, potentially affecting T-cell infiltration^33^. Consistent with this conclusion, our study found that the high-risk cohort presented an increased infiltration of activated CD4^+^/CD8^+^ T cell. Nonetheless, the prognosis was unfavorable in this group, potentially due to T-cell anergy via mechanisms involving GRAIL^34^. NPM1, implicated in various cellular activities, shows significant overexpression in hyperdiploid MM due to chromosome 5 gains, suggesting its key role in the pathogenesis of hyperdiploid MM^35^. Additionally, CRIP might be linked to intestinal zinc transport and myeloma bone disease severity^36^. Our findings reflected that C1orf56 served as a risk gene in MM, which is supported by the fact that C1orf56 is a proto-oncogene repressed by DNMT3B methylation. S100A6 expression was notably higher in primary MM patients than in controls, associating it with MM progression and intramedullary metastasis^37^. MEI1, presumed to be involved in meiosis I, is linked to gestational trophoblastic neoplasms. In our framework, elevated MEI1 expression contributes favorably to MM prognosis, but the exact mechanism requires further study. MGAT4A, encoding a pivotal glycosyltransferase, was observed to function as a protective gene in MM and positively impact prognosis in our study. The protective effect of MGAT4A has been observed in breast cancer, in which diminished expression is related to drug resistance^38^. In a preliminary search of the literature, we found no overlap between the 13 genes in this model and those in existing MM prognostic models. This may be related to the fact that there are currently no prognostic models for MM based on the genes of NETs, as well as our focus on the potentially possible significant prognostic contribution of the microenvironment. However, the mechanism through which the different myeloma cells stimulate the activation of NET-related genes in microenvironmental cells and the occurrence of adverse prognostic expression profiles needs to be further explored.

We noted that among the transcriptomic features of different cell types (malignant plasma cells vs. microenvironmental cells), six of the risk genes, C1orf56, CRIP1, GAPDH, HIST1H1C, RNF125, and S100A6, had a consistent trend of expression in the low- and high-risk groups differentiated by the NET-related prognostic model (Supplementary Fig. 3A, B). In order to clarify the difference in the contribution of these genes to prognosis in different cell types, we performed Quantitative Real-time PCR to detect the expression of the five genes (GAPDH as an internal reference gene) in myeloma and bone marrow stromal cells. The results (Supplementary Fig. 4A–E) suggest that three genes, CRIP1, HIST1H1C and RNF125, are significantly overexpressed in myeloma cell lines and may contribute to the poor prognosis in malignant plasma cell samples, whereas C1orf56 and S100A6 may contribute to the poor prognosis in microenvironmental cell samples.

The GO and KEGG annotation results revealed primary enrichments in cytoplasmic translation, ribosome assembly, and ribosome biogenesis (BP). Furthermore, significant annotations were noted in the CC of the cytosolic ribosome, ribosomal subunit, and ribosome. MF predominantly involved the structural constituent of ribosome, rRNA binding, and antigen binding. Other enrichments were identified in pathways such as COVID-19, ribosome, and protein processing in the endoplasmic reticulum. Many of these factors have been previously linked to the pathogenesis of ribosome biogenesis. Notably, the expression of genes related to ribosome biogenesis correlates with disease progression and prognosis in MM patients, suggesting potential therapeutic targets, including BRD9^39^.

GSEA facilitates the extraction of valuable insights from large-scale gene datasets, even with minimal fold changes. Utilizing GSEA of our gene datasets, we identified numerous gene sets significantly enriched within the MM group. Specifically, DNA replication gene overexpression in differentiated tissues could suggest a pathological state. In the MM dataset, this might imply an accelerated division of myeloma cells, a characteristic often observed in malignant tumors. The spliceosome, a complex molecular entity predominantly found in eukaryotic cell nuclei, primarily functions in excising introns and ligating exons during pre-mRNA processing, a critical step known as RNA splicing. This is essential for the optimal maturation of mRNA prior to its export for protein translation. Disruptions in spliceosome function can induce aberrant mRNA splicing, potentially producing dysfunctional proteins. Research by Hector H. Huang identified splicing interference as a novel aspect of the PI mechanism, unveiled further spliceosome modulation methods, and posited spliceosome targeting as a promising therapeutic approach for MM^40^. Given the likelihood that the DEGs we extracted came from cells from the BM microenvironment, common comorbidities of elderly such as Parkinson's disease and asthma may be confounding.

We chose the MM dataset with screened myeloma cells (GSE4581) for GSEA and GSVA analyses and obtained different results. Pathway co-enriched by malignant plasma cells and microenvironmental cells was DNA REPLICATION. Also, the difference was that malignant plasma cells were enriched to pathway properties of the tumor cell itself, such as the CELL CYCLE and PROTEASOME, whereas microenvironmental cells were enriched to the pathway of transcription and translation, such as SPLICEOSOME and RIBOSOME (Supplementary Fig. 5A–F). GSVA analyses were also suggestive of showing that myeloma cells and microenvironmental cells differ significantly in the pathways with the most significant differential expression in the low- and high-risk groups distinguished by our NET-related gene prognostic model (Supplementary Fig. 5G).

The dynamic interaction between myeloma cells and the BM microenvironment plays a pivotal role in malignant transformation, treatment response, and disease progression. Our comprehensive investigation of the prognostic-signature-based immune distinctions revealed that the high-risk group exhibited augmented infiltration of cells linked to adaptive immunity. Conversely, the low-risk group had a pronounced infiltration of cells associated with the innate immune system. These variations in cellular infiltration suggest a heightened propensity in the high-risk group to develop immune evasion through mechanisms such as immune resistance, exhaustion, and suppression^41^. The high-risk cohort showed increased infiltration of type 2 T helper cells (HR = 5.7, *p* = 0.013) and decreased infiltration of NK cells (HR = 0.089, *p* = 0.046). Both have been previously identified as significant adverse prognostic factors in MM^42^. Type 1 T helper cells generate IFN-γ, bolstering the cell-mediated immune response, while type 2 T helper cells, which produce IL-4, counteract this type 1 T helper cells response. Research by Faqing Tian et al. uncovered that myeloma cells could serve as antigen presenting cells (APCs), showcasing microbial antigens to type 2 T helper cells, which spurs their proliferation and thereby aids tumor progression via intimate Th2-myeloma cell interactions^43^. NK cells possess a spectrum of antitumor and immunomodulatory functions. A direct correlation exists between NK cell activity and disease-free survival in MM patients. Reduced NK cell activity aligns with advanced clinical stages, elevated LDH, heightened BM plasma cell infiltration, and increased β2 microglobulin levels^44^. Those MM patients exhibiting long-term disease stability displayed an expansion of NK cells^45^. In our study, this finding is consistent with those previous conclusions made by others. Additionally, our findings suggest that neutrophils in myeloma patients engage in aberrant interactions with NK cells via receptors such as HLA-E, which could account for the diminished NK cell presence in the high-risk cohort, consequently affecting prognosis. Therefore, devising immune therapies targeting NK cells, such as BCMA CAR-NK^46^, seems promising for the high-risk group. Evidently, these immune cellular infiltration disparities could be the underlying factors for the adverse prognosis observed in the high-risk group, with this group exhibiting markedly lower survival rates than their low-risk counterparts.

To elucidate the impact of immune cell infiltration in MM more profoundly, we employed ssGSEA for a thorough assessment of immune infiltration within MM contexts. Our analysis revealed that heightened infiltration of memory B cells, type 2 T helper cells, and activated CD4^+^ T cells might be intrinsically linked to the onset and progression of MM. Notably, ATF7IP2 demonstrated a significant association with memory B cells, while ATF7IP2, RNF125, and C1orf56 showed substantial correlations with type 2 T helper cells. Furthermore, ATF7IP2, C1orf56, and CPIP1 exhibited strong associations with activated CD4^+^ T cells. Conversely, MGAT4A and MEI1 displayed negative correlations with memory B cells. Within our model, ATF7IP2 emerges as the predominant gene contributing to a low-risk profile. This observation aligns with prior findings in studies on lung cancer^47^. Interestingly, ATF7IP2 bears positive correlations with diverse immune cell infiltrations, typically linked to unfavorable prognoses. This implies that ATF7IP2 might positively influence prognosis via alternative pathways. Such hypotheses necessitate further exploration to elucidate the intricate interplay between genes and immune cells, providing a direction for the in-depth exploration and supplementation of the potential molecular mechanism in MM.

Drug sensitivity prediction analysis suggests that high-risk patients might have heightened sensitivity to bortezomib treatment. This raises the possibility that primary induction therapy, which includes the PI bortezomib such as the VCD (bortezomib, cyclophosphamide, dexamethasone) regimen, may not fully explain the observed prognostic disparities between groups. Moreover, venetoclax appears to be more effective in the high-risk cohort. Its use has been chiefly confined to patients with cytogenetic abnormalities characterized by the t(11:14) translocation, which results in the IgH/CCND1 fusion gene^48^. Notably, our prognostic model includes the CCND1 gene, which is believed to act as a protective factor. This is consistent with the favorable prognosis in the myeloma subgroup with the IGH/CCND1 fusion gene, implying the venetoclax might be beneficial for high-risk patients with elevated CCND1 expression. Additionally, Selinexor, a selective nuclear export inhibitor, has shown significant efficacy against relapsed and refractory MM^49^. Given its capacity to inhibit human NET formation in vitro^50^, selinexor may offer potential as a salvage therapy for high-risk patients. Furthermore, AZD7762, an ATP-competitive checkpoint kinase inhibitor, augments checkpoint termination and bolsters DNA-focused treatments. For refractory high-risk patients, its combined administration with cytotoxic agents or immune checkpoint inhibitors^51^ could be advantageous. In contrast, for low-risk patients, conventional treatments yield moderate drug sensitivity. For those unresponsive to standard treatments, small molecule drugs such as the MAPK inhibitor Doramapimod and the ATM inhibitor KU-55933 might be viable options. The results of drug sensitivity prediction analysis provide a possible direction for MM therapy, but all of speculations need in-depth experiments to verify.

This study presents several limitations warranting acknowledgment. First, the NET-dependent risk signature was developed based on a limited sample of MM patients sourced from the GEO databases. To validate the predictive relevance of this prognostic signature, expansive prospective clinical studies are necessary. Furthermore, the NET-dependent risk signature was derived exclusively from bioinformatics analysis, necessitating further empirical research to substantiate the findings. It is also important to note that our prognostic model focuses mainly on the bone marrow microenvironment of MM, and has not yet taken into consideration the cytogenetic characteristics of the myeloma cells themselves which are the most important prognostic indicator of MM. The relationship between cytogenetics and this NET-related gene prognostic model needs to be further explored. NETs are likely to be associated with tumor growth, extramedullary metastasis and thrombosis^12^ in MM, and endothelial autophagy^52^ may be involved and play important roles. This is well worth exploring in depth. Unfortunately, we could not annotate endothelial cells in the analyzed dataset (GSE223060), which further may be resolved by retaining bone marrow biopsies instead of bone marrow fluid for single-cell sequencing.

Our study demonstrates that neutrophils, monocytes, and macrophages exhibit NET activity in MM. Subsequently, we identify an anomalous communication pathway between neutrophils and NK cells in MM. Ultimately, we present an innovative prognostic signature derived from NET-related genes for MM patients. This signature proficiently forecasts prognosis and has the potential to pave the way for further therapeutic exploration.

## Methods

### Bulk transcriptome data acquisition and preprocessing

All data utilized in this study are publicly available and primarily sourced from the Gene Expression Omnibus (GEO, https://www.ncbi.nlm.nih.gov/geo/). The MM genome-wide expression profiles were retrieved using the R package 'GEOquery' from the GEO database. We focused on the characterization of NET-active cells in the bone marrow microenvironment and therefore chose a MM transcriptome dataset that was subtracted from malignant plasma cell signaling (GSE136337). This research incorporated GSE136337, which comprises 424 tumor samples, and adhered to the data access policies of the respective database.

### Single-cell sequencing data download and processing

The GEO database houses a vast array of single-cell sequencing data. For this research, we accessed an MM single-cell sequencing dataset, GSE223060, from the GEO database, encompassing 41 disease samples and 19 normal samples. We imported the raw data from GSE223060 using the Seurat package for R (version 4.2.0)^53^. The dataset underwent preliminary filtering based on several criteria to ensure that only high-quality cells were used for the subsequent analyses: (1) Exclusion of genes found in fewer than 1 cell. (2) Retention of cells with gene expression ranging from 200 to 5000 for the reason that too few genes expressed reflect possible debris or low-activity cells, while too many genes expressed reflect the presence of cell doublet or multiplet. (3) Retention of cells with mitochondrial gene percentages below 20% for the elimination of low-activity or dying cells. (4) Retention of cells with unique molecular identifier (UMI) counts between 1000 and 10,000 for the reason same as criteria 2). The condition of cells before and after filtration was shown by violin plots (Supplementary Fig. 6A, B). The data were normalized with the 'normalizedata' function in Seurat. Following normalization, we pinpointed the highly variable genes in single cells, taking into account the correlation between average expression and dispersion. We conducted a principal component analysis (PCA) using significant principal components (PCs) for graph-based clustering. Batch correction for the different samples was performed by the ‘RunHarmony’ function in the R package harmony (version 0.1.0), and the batch effect was well removed. During clustering, the FindClusters function was utilized, which implements the shared nearest neighbor (SNN) modularity optimization-based clustering algorithm on 30 PC components at a resolution of 0.3 where the clustering was most stable (Supplementary Fig. 7), resulting in 22 clusters. We executed uniform manifold approximation and projection (UMAP) using the 'Runumap' function, visualizing cell clusters via UMAP-1 and UMAP-2 coordinates. To discern differentially expressed genes (DEGs) within each cluster, we engaged the FindAllMarkers function from Seurat on the normalized gene expression data. Following this, we identified the cell clusters using cell type-specific biomarkers and assessed the proportions of the various cell types.

### Neutrophil extracellular trap-related gene score

Using the AUCell R package^54^, pathways for each cell were scored based on gene set enrichment analysis (GSEA). The gene set of neutrophils was derived from Şenbabaoğlu et al.^55^. NETosis-related genes were largely informed by a review article outlining advances in the study of NETs in immunity and various diseases^11^. In summary, we adopted the 69 genes identified by Zhang and colleagues as initial biomarkers for NETs characterization training^30^ (Table S12). Scores were derived from the area under the curve (AUC) values of the selected 69 NET-related genes. By ranking the gene expression of each cell, we estimated the proportion of highly expressed genes in each cell. Cells with more genes from the set exhibited higher AUC values. We employed the ‘AUCell_exploreThresholds’ function to determine the threshold for recognizing cells with active gene sets. Subsequently, the AUC scores of individual cells were visualized on the UMAP embedding using the ‘ggplot2’ R package (Version 3.3.5), highlighting the active clusters.

### Constructing single-cell trajectory in pseudo-time

We performed pseudo-time analysis using Monocle 2^56^, leveraging reverse graph embedding based on a user-specified gene list to produce a pseudo-time plot that captures both branched and linear differentiation trajectories. For this analysis targeting the neutrophil active cells, raw count data underwent normalization by calculating the size factors essential for trajectory inference. Only genes exhibiting high dispersion (empirical dispersion/dispersion fit ≥ 1) and significant expression (mean expression ≥ 0.1) were selected to construct the pseudo-time trajectory^57^. We employed the default parameters of the DDRTree algorithm for this purpose. The branching events in the trajectories were further examined using branched expression analysis modeling (BEAM) within Monocle 2. This approach aids in pinpointing genes demonstrating noteworthy branch-dependent expression^56^. Monocle 2 was also used to visually represent these branch-dependent expression patterns in a heatmap format.

### Cell communication analysis and ligand-receptor expression

Cell–cell communication analysis evaluates the expression of ligand-receptor pairs across various cell types, highlighting specific signaling pathways^58^. CellChat discerns both the afferent and efferent communication patterns of each cell type, quantifies the cellular communication pathway, and computes the information flow for each signaling pathway or the intercellular communication probability^59^. In our research, we employed CellChat to investigate single-cell samples. Utilizing CellChat (version 1.1.3), we assessed the intercellular communication across cell types within each MM sample by incorporating the standardized scRNA-seq data after Seurat package processing. We carried out a detailed analysis of the cellular communication signals in MM, focusing specifically on neutrophils to ascertain the intensity of each signaling pathway and further selecting distinct communication pathways for visualization. Our CellChat analyses maintained the default software parameters, setting *p* ≤ 0.05 as the significance threshold, with the adjusted* p* value corrected via the Benjamini and Hochberg (BH) method.

### GO and KEGG pathway enrichment analysis

Gene Ontology (GO)^60^ enrichment analysis encompasses biological process (BP), molecular function (MF), and cellular component (CC) categories. The Kyoto Encyclopedia of Genes and Genomes (KEGG)^61^ serves as a bioinformatics tool to identify notably altered metabolic pathways enriched within the gene list. Using the 'clusterProfiler' R package (version 4.2.2)^62^, we conducted both GO and KEGG enrichment analyses on NET-related DEGs in MM, setting a significance threshold at* p* < 0.05.

### Development and validation of the prognostic system

For difference analysis of the transcriptome data (GSE136337) of the MM samples and normal controls, we used the 'limma' R package (version 3.50.0) for difference analysis and the BH method for multiple calibration. To determine the prognostic significance of the NET-related DEGs, we conducted a univariate Cox hazard analysis, assessing the associations between each gene and overall survival (OS) within the tumor cohorts. Genes correlated with survival, using a cutoff *p* value of 0.05, were selected for subsequent analysis. Tumor samples with accompanying clinical data were stratified into a training set (n = 291) and a verification set (n = 129) at a 7:3 ratio. We employed the LASSO Cox regression model (R package 'glmnet'^63^) with 10-fold cross-validation to refine the list of candidate genes and formulate the prognostic model. The penalty parameter (λ) was determined based on minimum criteria. The risk score was computed using the following formula:

$${\text{riskScore}} = \sum\limits_{i = 1}^{n} {{\text{Coef}}\left( {{\text{gene}}_{i} } \right)} *{\text{Expression}}\left( {{\text{gene}}_{i} } \right)$$

(Coef (gene_i_): coefficients, Expression (gene_i_): gene expression level).

Patients in the training group were categorized into low- and high-risk subgroups based on the median risk score. The Kaplan–Meier method was used to generate survival curves for prognostic evaluation, while the statistical significance between the groups was determined using log-rank tests. The efficacy of the prediction model was assessed through receiver operating characteristic (ROC) curves. AUC values, which typically vary between 0.5 and 1, serve as indicators of the model's performance, with values closer to 1 denoting optimal efficacy. For validation, the verification groups were similarly divided into risk subgroups, and these categories were subsequently compared to authenticate the gene model.

### Quantitative real-time polymerase chain reaction (RT-qPCR)

We performed RT-qPCR to verify the differential expression of risk genes in myeloma and bone marrow stromal cell lines, and the raw data was shown in Table S13. The main experimental procedures and methods were presented in Supplementary File 15.

### Construction and verification of the nomogram

Clinical data, encompassing survival status, survival duration, age, and sex, were sourced from the GEO cohort. These variables were integrated into our regression model with the risk score. Both univariate and multivariable Cox regression models facilitated the analysis. A nomogram was constructed to forecast the 1-, 3-, and 5-year survival probabilities, incorporating the risk score as a prognostic factor. By amalgamating prognostic indicators with clinical data, the nomogram was executed using the ‘RMS’ R package. The efficacy of the risk score model and the nomogram were assessed using time-dependent ROC curves.

### Gene set enrichment analysis

GSEA^64^ is a computational technique that ascertains whether an a priori defined gene set exhibits statistically significant, concordant variations between two biological conditions. In this study, the differential expression between low- and high-risk groups was analyzed using the 'limma' R package^65^. The fold change (FC) in gene expression between these groups was determined. Subsequently, GSEA was conducted with the 'clusterProfiler' R package (version 4.2.2), utilizing an ordered list of genes based on their log2FC values. The analysis underwent 1,000 gene set permutations. The reference gene collection was selected as c2.cp.kegg.v7.5.1.symbols from the Molecular Signatures Database (MSigDB)^64,66,67^ Collections. A gene set was deemed to have significant enrichment if it had an adjusted* p* value of less than 0.05.

### Gene set variation analysis

To explore the differences in biological function between control and MM samples, we employed gene set variation analysis (GSVA) using "c2.cp.kegg.v7.5.1.symbols" through the R package 'GSVA (version 1.42.0)'. Visualization of the results was achieved using the 'pheatmap' R package (version 1.0.12).

### Immune infiltration analysis

Single-sample gene set enrichment analysis (ssGSEA)^68^, a derivative of GSEA, computes enrichment scores for each combination of sample and gene set. Each ssGSEA enrichment score indicates the coordinated up- or downregulation of genes within a specific gene set for an individual sample. Unlike traditional GSEA, which calculates enrichment scores for groups of samples (e.g., control vs. disease) and gene sets (e.g., pathways), ssGSEA provides a score for each sample-gene set pairing.

Using the 28 types of immune cells sourced from the TISIDB (Tumor and Immune System Interactions Database) (http://cis.hku.hk/TISIDB/index.php)^68^ (Table S14), such as activated CD8^+^ T cells and natural killer (NK) cells, the relative enrichment score for each immunocyte was derived from the gene expression profiles of MM samples. Differences in the infiltration levels of these immune cells between the low- and high-risk groups were visualized using the 'ggplot2' R package (version 3.3.6)^69^.

### Assessment of drug susceptibility

Using the half-maximal inhibitory concentration (IC50) from the Genomics of Drug Sensitivity in Cancer (GDSC) database (https://www.cancerrxgene.org/)^70^ and clinical gene expression data, we employed the 'oncoPredict (version 0.2)' R package^71^ to predict the potential therapeutic drug sensitivity for MM patients in both risk subgroups.

### Statistical analysis

The Wilcoxon rank-sum test was employed to assess the relationship between continuous variables in the low- and high-risk groups. Proportional differences were assessed using the chi-square test or Fisher's exact test. Kaplan–Meier survival curves were generated using the ‘ggsurvplot’ function from the 'survminer' package in R, and significant differences were evaluated with the log-rank test. We used LASSO-Cox regression analyses to develop signature genes and produced ROC and time-ROC curves to evaluate predictive performance. Statistical significance was set at a two-sided* p* value < 0.05. All analytical procedures were performed using R software (version 4.1.2).

### Supplementary Information

Supplementary Information 1.

Supplementary Information 2.

Supplementary Information 3.

Supplementary Information 4.

Supplementary Information 5.

Supplementary Information 6.

Supplementary Information 7.

Supplementary Information 8.

Supplementary Information 9.

Supplementary Information 10.

Supplementary Information 11.

Supplementary Information 12.

Supplementary Information 13.

Supplementary Information 14.

Supplementary Information 15.

Supplementary Information 16.

Supplementary Information 17.

Supplementary Information 18.

Supplementary Information 19.

Supplementary Information 20.

Supplementary Information 21.

Supplementary Information 22.

Supplementary Information 23.

## Data Availability

The datasets generated and/or analyzed during the current study are available in the GEO dataset (https://www.ncbi.nlm.nih.gov/geo/). The single-cell dataset GSE223060: https://www.ncbi.nlm.nih.gov/geo/query/acc.cgi?acc=GSE223060. The transcriptomics dataset GSE136337: https://www.ncbi.nlm.nih.gov/geo/query/acc.cgi?acc=GSE136337. The transcriptomics dataset GSE4581: https://www.ncbi.nlm.nih.gov/geo/query/acc.cgi?acc=GSE4581. This article and supplemental material included all the data generated during this study. For further inquiries, please contact the corresponding author.

## Abbreviations

MM: Multiple myeloma
BM: Bone marrow
SEER: Surveillance, Epidemiology, and End Results
ISS: International Staging System
LDH: Lactic dehydrogenase
TME: Tumor microenvironment
VEGF: Vascular endothelial growth factor
TGF-β: Transforming growth factor beta
IL-6: Interleukin-6
MDSCs: Myeloid-derived suppressor cells
NET: Neutrophil extracellular trap
PAD4: Peptidylarginine deiminase 4
ALL: Acute lymphoblastic leukemia
scRNA: Single-cell RNA
GEO: Gene Expression Omnibus
UMI: Unique molecular identifier
PCA: Principal component analysis
PC: Principal component
SNN: Shared nearest neighbor
UMAP: Uniform manifold approximation and projection
DEG: Differentially expressed gene
GSEA: Gene set enrichment analysis
AUC: Area under the curve
BEAM: Branched expression analysis modeling
BH: Benjamini and Hochberg
GO: Gene ontology
BP: Biological process
MF: Molecular function
CC: Cellular component
KEGG: Kyoto Encyclopedia of Genes and Genomes
OS: Overall survival
LASSO: Least absolute shrinkage and selection operator
ROC: Receiver operating characteristic
FC: Fold change
MSigDB: Molecular Signatures Database
GSVA: Gene set variation analysis
ssGSEA: Single-sample gene set enrichment analysis
TISIDB: Tumor and Immune System Interactions Database
NK: Natural killer
IC50: Half-maximal inhibitory concentration
GDSC: Genomics of Drug Sensitivity in Cancer
DCs: Dendritic cells
pDCs: Plasmacytoid dendritic cells
MIF: Macrophage migration inhibitory factor
LCK: Lymphocyte specific protein tyrosine kinase
HLA: Human leukocyte antigen
MHC: Major histocompatibility complex
rRNA: Ribosomal RNA
NES: Normalized enrichment score
FDR: False discover rate
PI: Proteasome inhibitor
IFN-γ: Interferon gamma
IL-4: Interleukin-4
APCs: Antigen presenting cells
BCMA: B cell maturation antigen
CAR: Chimeric antigen receptor
VCD: Bortezomib, cyclophosphamide, dexamethasone
MAPK: Mitogen-activated protein kinase
ATM: Ataxia telangiectasia mutated protein
