# Supplementary figures and images for: A neutrophil extracellular trap-related risk score predicts prognosis and characterizes the tumor microenvironment in multiple myeloma
Source: Sci Rep. 2024 Jan 27;14:2264. doi: 10.1038/s41598-024-52922-7 (PMC10817968; doi:10.1038/s41598-024-52922-7)

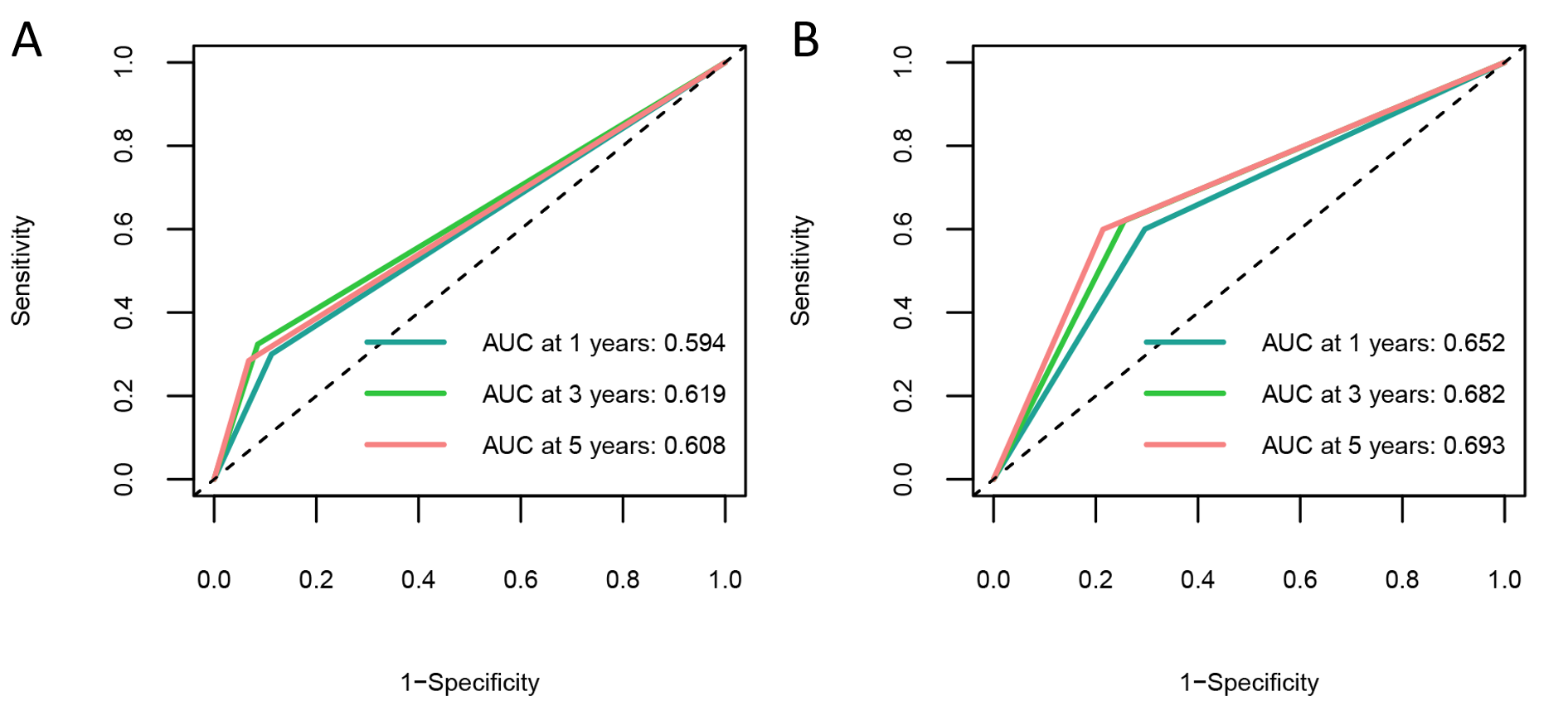

Supplement: Supplementary file 1 — Supplementary Information 1. [file 41598_2024_52922_MOESM1_ESM.tif]

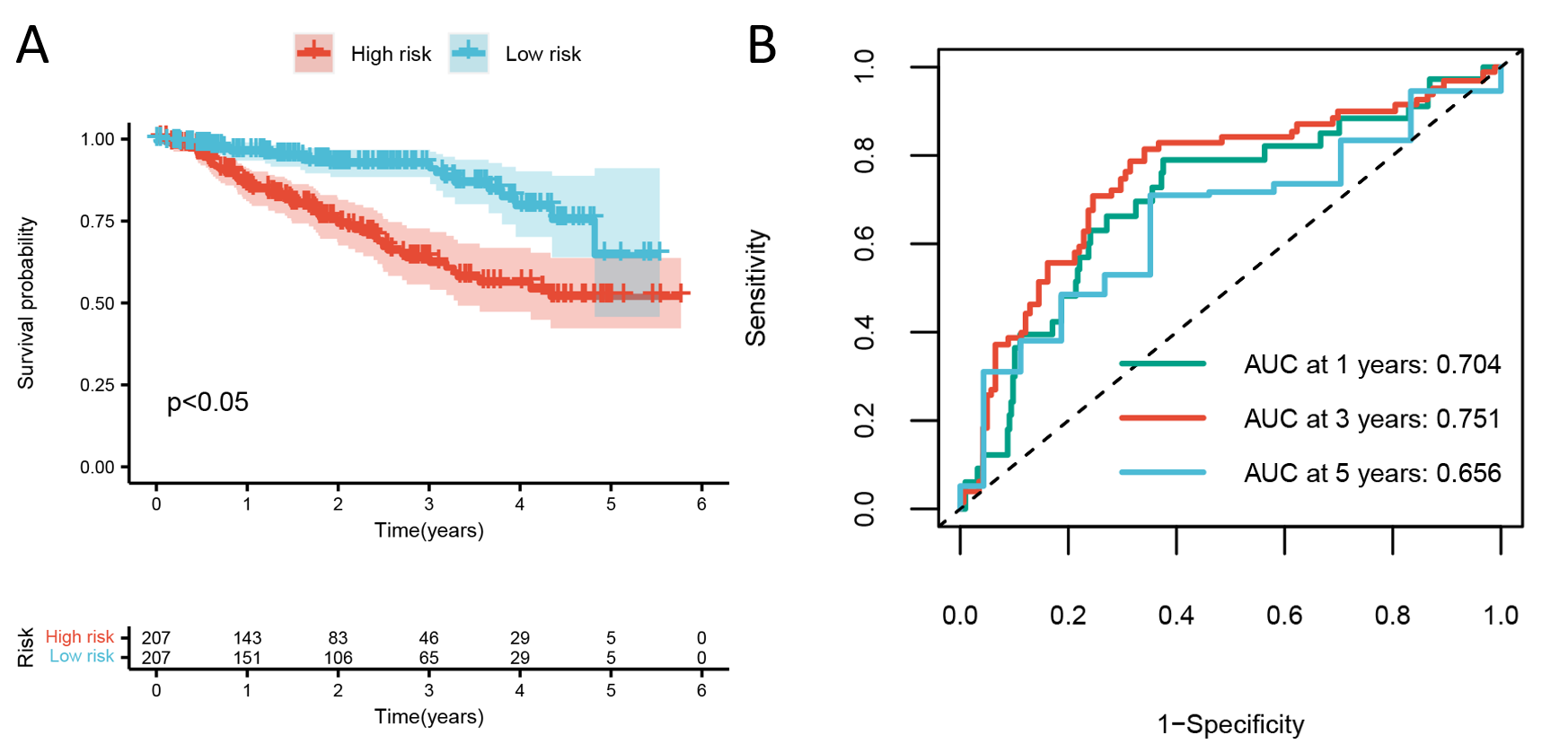

Supplement: Supplementary file 2 — Supplementary Information 2. [file 41598_2024_52922_MOESM2_ESM.tif]

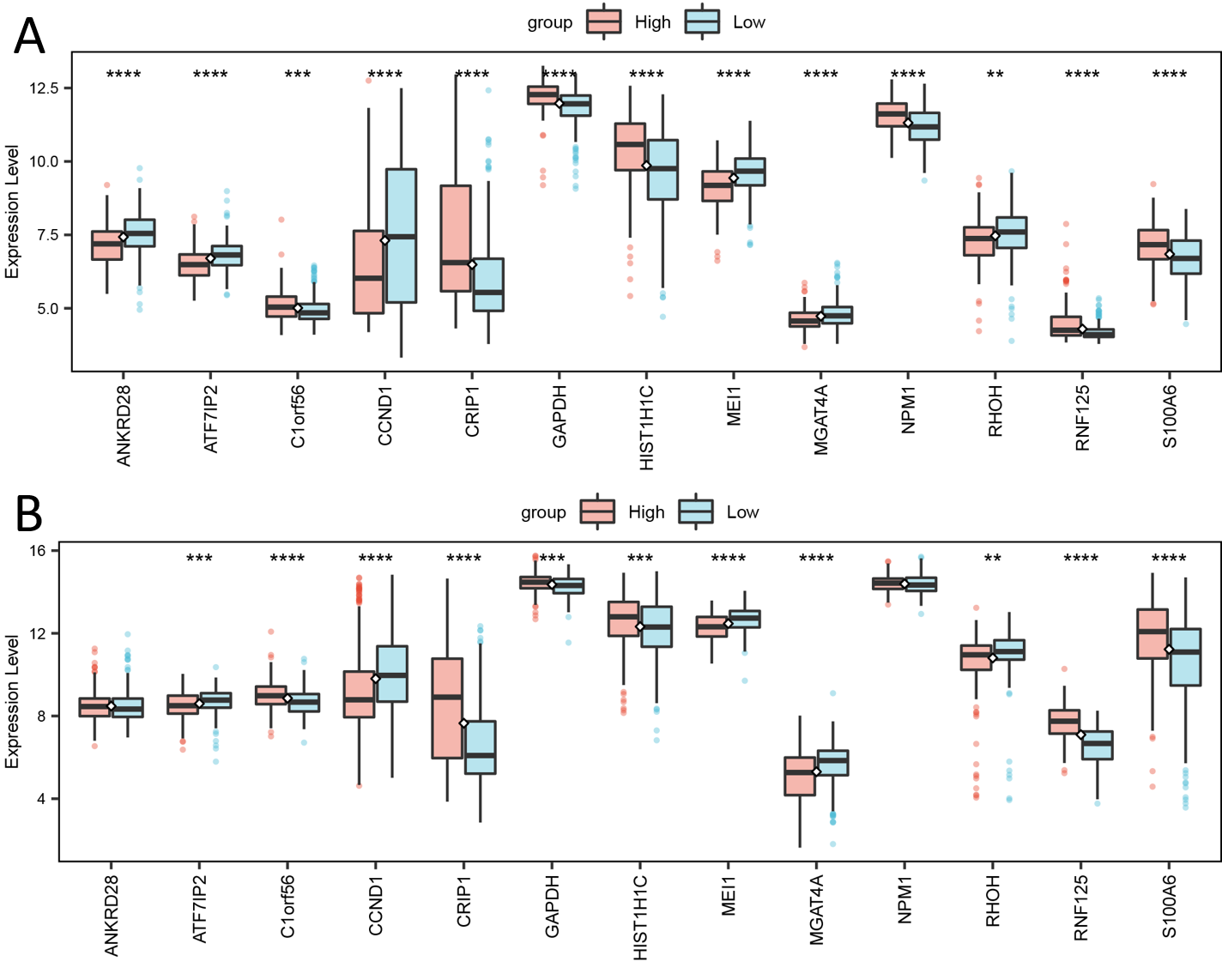

Supplement: Supplementary file 3 — Supplementary Information 3. [file 41598_2024_52922_MOESM3_ESM.tif]

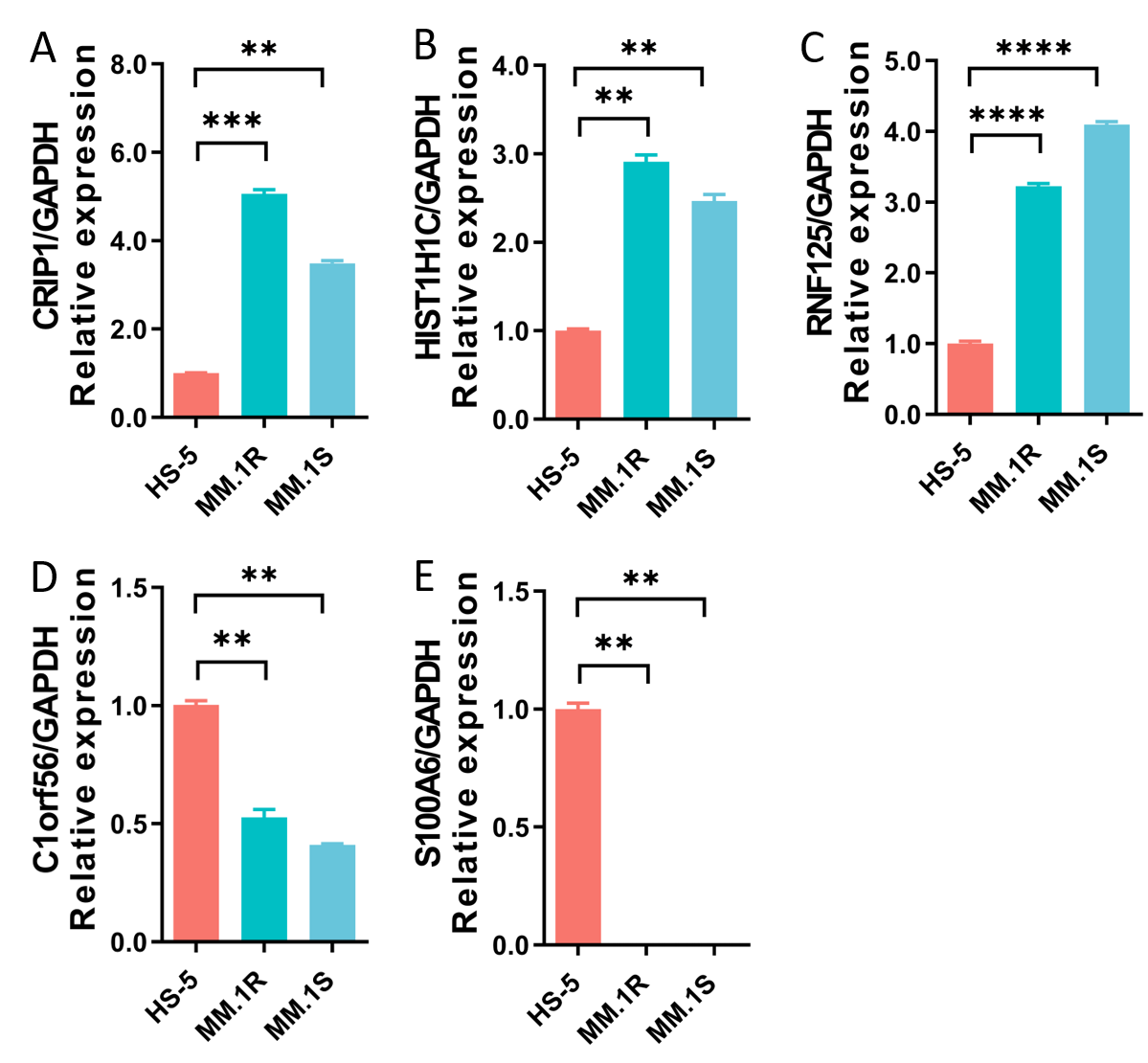

Supplement: Supplementary file 4 — Supplementary Information 4. [file 41598_2024_52922_MOESM4_ESM.tif]

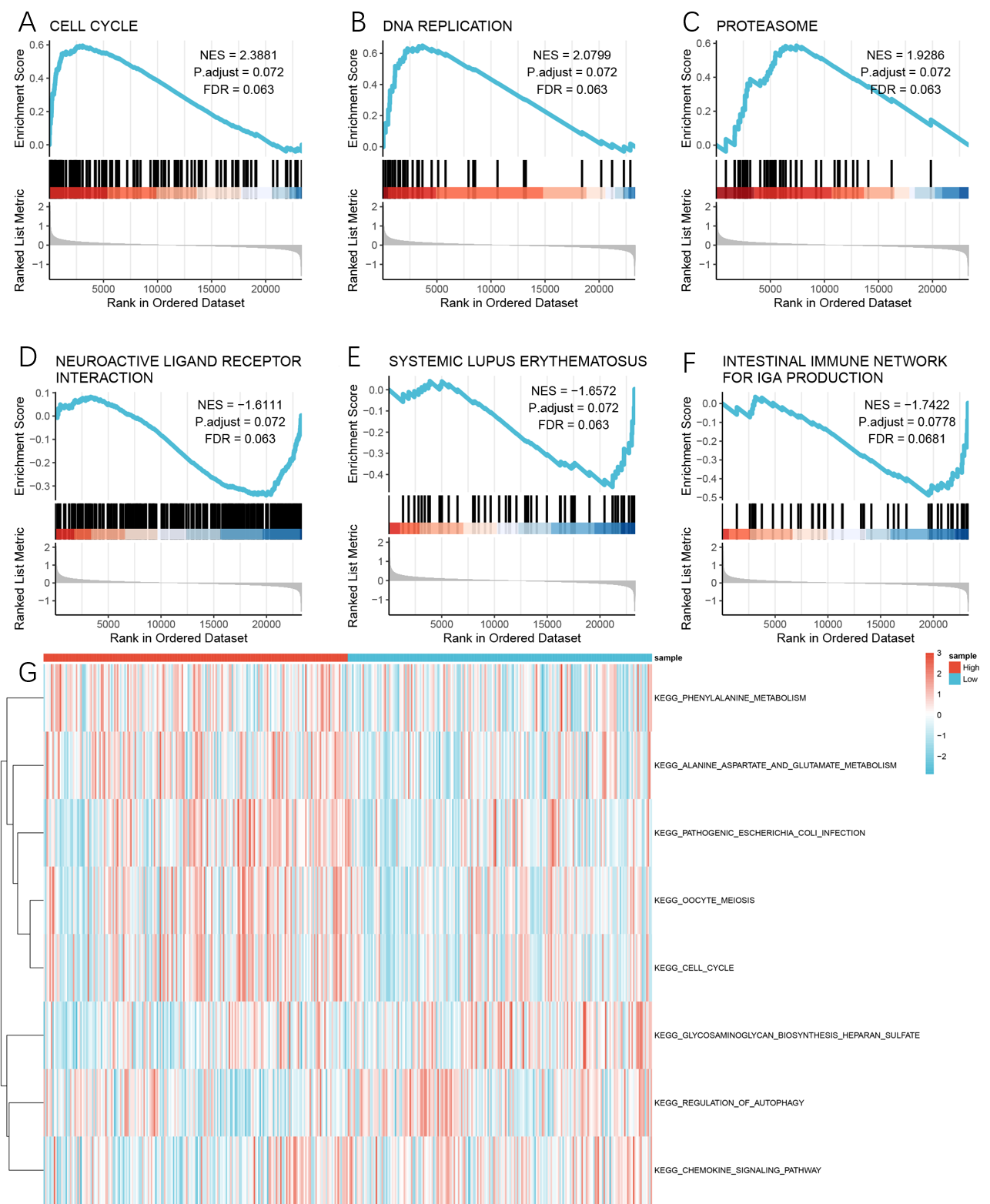

Supplement: Supplementary file 5 — Supplementary Information 5. [file 41598_2024_52922_MOESM5_ESM.tif]

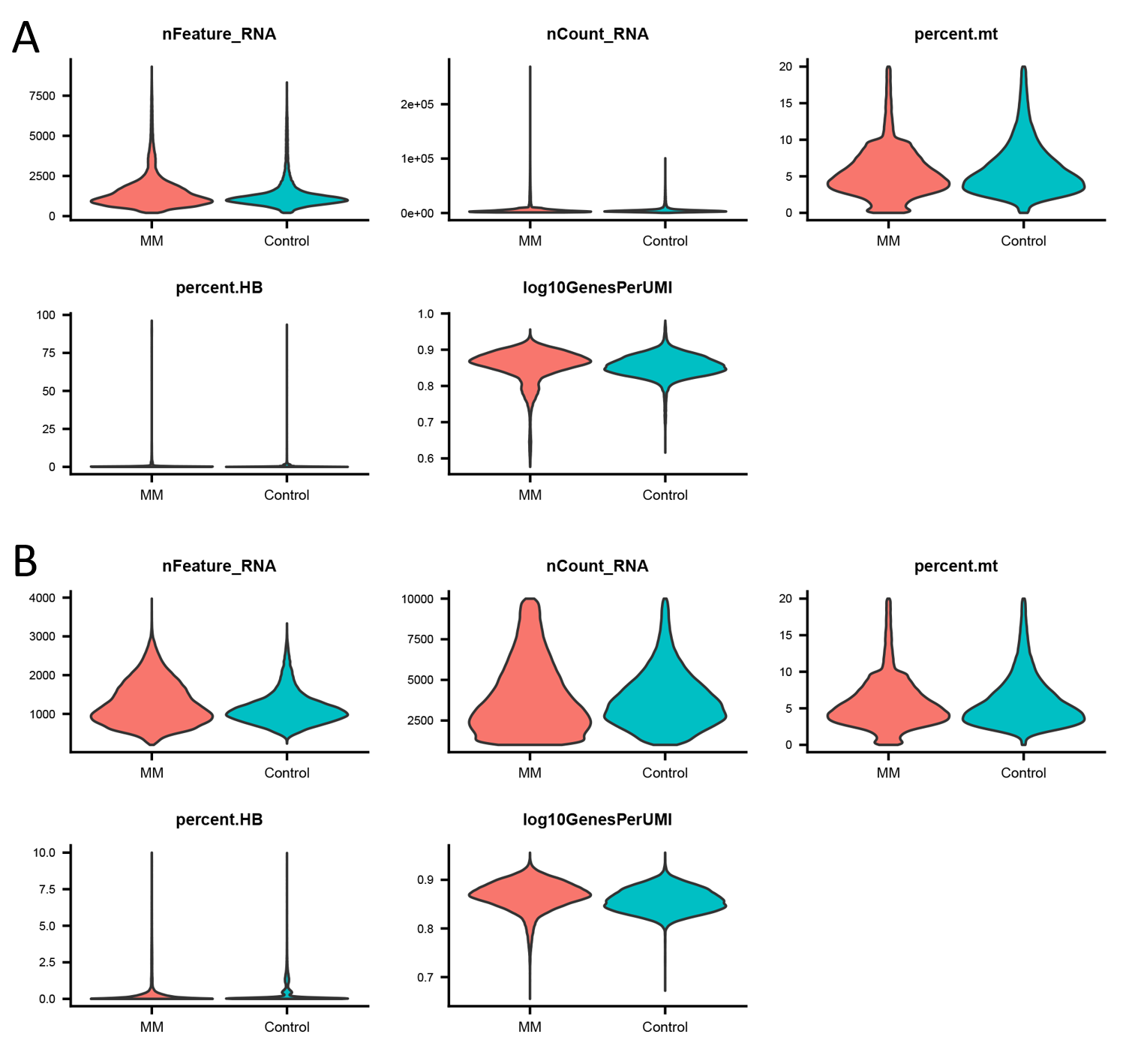

Supplement: Supplementary file 6 — Supplementary Information 6. [file 41598_2024_52922_MOESM6_ESM.tif]

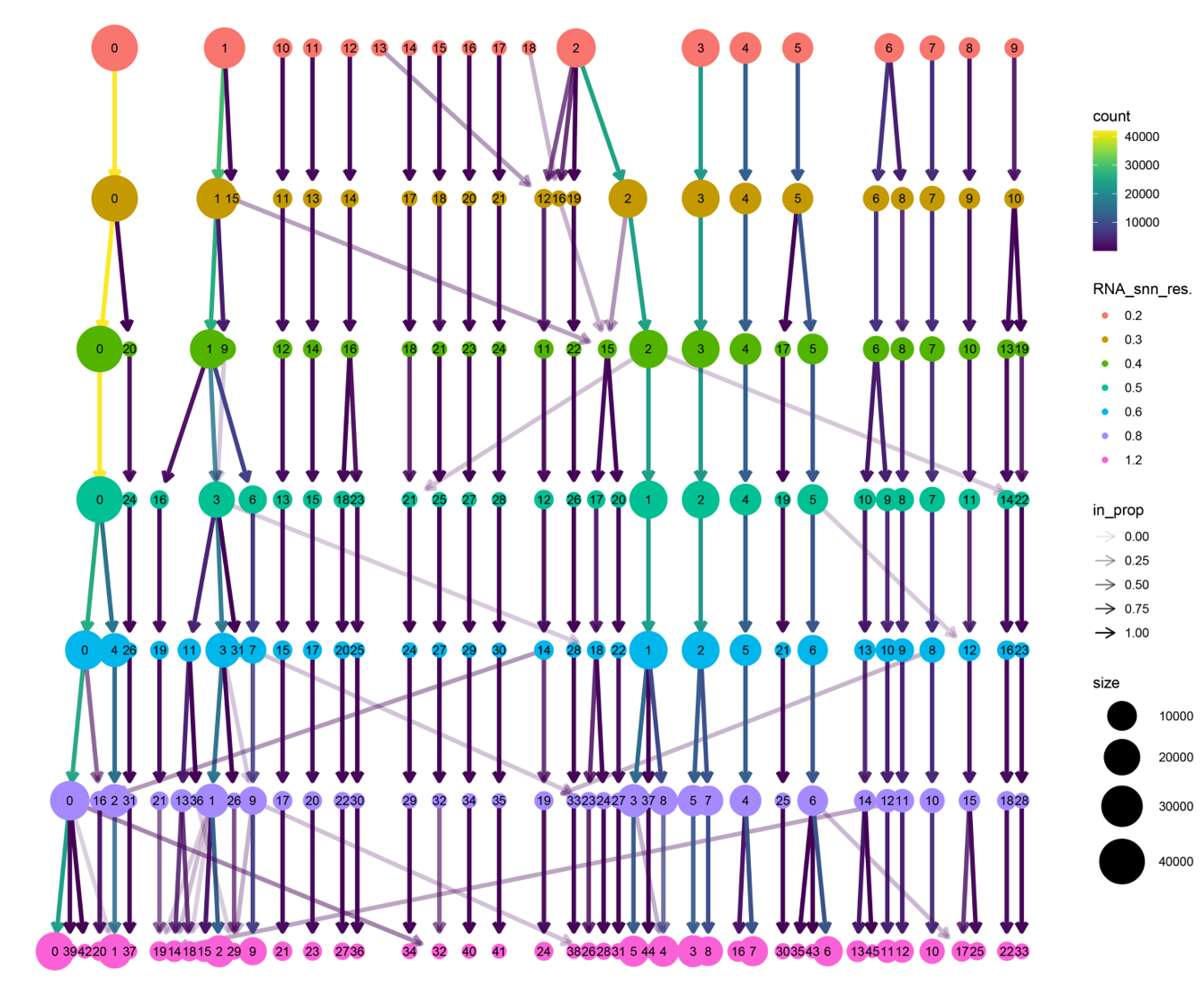

Supplement: Supplementary file 7 — Supplementary Information 7. [file 41598_2024_52922_MOESM7_ESM.tif]
