# Supplementary material for: A neutrophil extracellular trap-related risk score predicts prognosis and characterizes the tumor microenvironment in multiple myeloma
Source: Sci Rep. 2024 Jan 27;14:2264. doi: 10.1038/s41598-024-52922-7 (PMC10817968; doi:10.1038/s41598-024-52922-7)
Supplement: Supplementary file 8 — Supplementary Information 8. [file 41598_2024_52922_MOESM8_ESM.pdf]

Supplementary figure legends

Supplementary Figure 1. 1-, 3-, and 5-year time-dependent ROC curves in GSE136337. (A) The GEP70 model. (B) This NET-related model.

Supplementary Figure 2. Validation of the NET-related model in datasets GSE4581. (A) The survival curve of patients in low- and high-risk groups, respectively. (B) 1-, 3-, and 5-year time-dependent ROC curves.

Supplementary Figure 3. Expression levels of 13 genes in NET-related prognostic model between low- and high-risk groups. (A) In GSE136337. (B) In GSE4581.

Supplementary Figure 4. Expression level of risk genes in myeloma and bone marrow stromal cell lines. (A) CRIP1. (B) HIST1H1C. (C) RNF125. (D) C1orf56. (E) S100A6. (*$0.005<p<0.05$; **$0.0005<p<0.005$; *** $0.0001<p<0.0005$; ****$p<0.0001$)

Supplementary Figure 5. GSEA and GSVA of significantly enriched pathways in GSE4581. (A-F) CELL CYCLE, DNA REPLICATION, PROTEASOME, NEUROACTIVE LIGAND RECEPTOR INTERACTION, SYSTEMIC LUPUS ERYTHEMATOSUS and INTESTINAL IMMUNE NETWORK FOR IGA PRODUCTION. (G) GSVA of significantly enriched pathways.

Supplementary Figure 6. The condition of cells. (A) Before filtration. (B) After filtration.

Supplementary Figure 7. Clustering at different resolutions by clustree.
