# Supplementary material for: A neutrophil extracellular trap-related risk score predicts prognosis and characterizes the tumor microenvironment in multiple myeloma
Source: Sci Rep. 2024 Jan 27;14:2264. doi: 10.1038/s41598-024-52922-7 (PMC10817968; doi:10.1038/s41598-024-52922-7)
Supplement: Supplementary file 12 — Supplementary Information 12. [file 41598_2024_52922_MOESM12_ESM.pdf]

"gene_name"
"ISG15"
"RPL11"
"IFI6"
"RPS8"
"IFI44L"
"RPL5"
"CTSS"
"S100A11"
"S100A6"
"RPS27"
"FCRL5"
"MNDA"
"SLAMF7"
"FCER1G"
"FCMR"
"RPS7"
"SDC1"
"RHOB"
"CDC42EP3"
"RHOQ"
"RPS27A"
"PLEK"
"GNLY"
"IGKC"
"DUSP2"
"RPL31"
"CXCR4"
"BAZ2B"
"FRZB"
"STAT1"
"STK17B"
"EEF1B2"
"RPL37A"
"ITM2C"
"ARL4C"
"RPL32"
"RPL15"
"RPSA"
"RPL14"
"GPX1"
"MANF"
"RPL29"
"RPL24"
"ZBTB20"
"ZBTB38"
"SEC62"
"TNFSF10"
"ST6GAL1"
"RPL35A"
"LAP3"
"RPL9"
"RHOH"
"JCHAIN"
"RPL34"
"SNHG8"
"ANXA5"

"RPS3A"
"RPL37"
"RPS23"
"VCAN"
"TCF7"
"MZB1"
"CD74"
"RPS14"
"NPM1"
"CANX"
"MGAT1"
"IRF4"
"TXNDC5"
"CD83"
"HIST1H1C"
"LTB"
"AIF1"
"CLIC1"
"HLA-DRA"
"HLA-DQB1"
"HLA-DPA1"
"HLA-DPB1"
"RPS18"
"RPS10"
"RPL10A"
"CPNE5"
"EEF1A1"
"PRDM1"
"RPS12"
"GLCCI1"
"TOMM7"
"HSPB1"
"ARPC1B"
"DNAJB9"
"TRBC2"
"SAT1"
"CYBB"
"TIMP1"
"CFP"
"RBM3"
"PIM2"
"RPS4X"
"SEPT6"
"SSR4"
"RPL10"
"SARAF"
"RPS20"
"LY96"
"TPD52"
"RPL30"
"RPS6"
"MLLT3"
"TXN"
"RPL35"
"RPL7A"
"FCN1"
"IFITM1"

"IFITM3"
"RPLP2"
"RPL27A"
"CD59"
"SPI1"
"MS4A7"
"MS4A1"
"AHNAK"
"NEAT1"
"GSTP1"
"CCND1"
"SPCS2"
"RPS3"
"BIRC3"
"POU2AF1"
"CD3E"
"CD3G"
"RPS25"
"DNAJC1"
"MAP3K8"
"PSAP"
"RPS24"
"EIF3A"
"CCND2"
"GAPDH"
"C12orf57"
"PTPN6"
"CLEC2D"
"ARHGDIB"
"LDHB"
"FKBP11"
"TUBA1A"
"ITGB7"
"PFDN5"
"CD63"
"RPL41"
"RP11-1143G9.4"
"BTG1"
"CKAP4"
"RPL6"
"OAS1"
"RPLP0"
"DYNLL1"
"RPL21"
"EPSTI1"
"RPS29"
"NPC2"
"FOS"
"WARS"
"IGHG1"
"IGHG3"
"IGHM"
"ANXA2"
"RPL4"
"RPS2"
"IL32"
"TNFRSF17"

"RPS15A"
"COTL1"
"COX4I1"
"CYBA"
"RPL13"
"YWHAE"
"XAF1"
"RNASEK"
"GABARAP"
"CD68"
"TNFRSF13B"
"RPL23A"
"RPL19"
"GRN"
"CLTC"
"CD79B"
"PECAM1"
"RPL38"
"SEC11C"
"LMAN1"
"CST3"
"ZFAS1"
"RPS21"
"CIRBP"
"RPS15"
"AES"
"RPL36"
"RPS28"
"TMEM205"
"BST2"
"RPL18A"
"IFI30"
"TYROBP"
"ZFP36"
"RPS16"
"BLVRB"
"CD79A"
"POU2F2"
"RPL18"
"FTL"
"RPL13A"
"FCGRT"
"NOSIP"
"RPS5"
"DERL3"
"XBP1"
"PIK3IP1"
"RPL3"
"MEI1"
"TYMP"
"MX1"
"RCAN3"
"FCGR3A"
"CX3CR1"
"IL7R"
"LST1"
"CPVL"

"FGL2"
"TRBC1"
"CD3D"
"ETS1"
"KLRB1"
"CD69"
"CLEC7A"
"TRAC"
"SERPINA1"
"BCL11B"
"IGHGP"
"CD7"
"GZMM"
"MYO1F"
"LGALS2"
"TENT5C"
"FYB1"
"NOP53"
"CD40"
"BMI1"
"CALR"
"MT-ND6"
"PDK1"
"ERLEC1"
"HIST1H4C"
"C1orf56"
"CRELD2"
"ATF7IP2"
"PPIB"
"LEPROTL1"
"IGLC2"
"SEPT7"
"FOXP1"
"MAF"
"SNRPD2"
"PIK3R1"
"HSP90B1"
"DNAJB1"
"HIST1H1D"
"MGAT4A"
"PPP1R10"
"CRIP1"
"PRDX4"
"SRPRB"
"LY6E"
"FKBP2"
"EPB41L4A-AS1"
"RBM38"
"RPS26"
"CREB3L2"
"MTRNR2L12"
"SDF2L1"
"ELL2"
"ERN1"
"KCNQ1OT1"
"SPCS1"
"TP53INP1"

"RACK1"
"CLPTM1L"
"SF1"
"IGHG4"
"PDIA4"
"RNF125"
"DST"
"COMMD3"
"ATP5MC2"
"KRTCAP2"
"IGHG2"
"C6orf48"
"HSPA5"
"TMED9"
"CD52"
"PDIA6"
"RPL36A"
"FAM107B"
"CYTOR"
"RABAC1"
"SELENOS"
"IGLC3"
"MT-ND5"
"MYDGF"
"CA1"
"HERPUD1"
"ANKRD28"
"RPS17"
"HBB"
"GLTSCR2"
"CFLAR"
"IGHA1"
