# Supplementary material for: A neutrophil extracellular trap-related risk score predicts prognosis and characterizes the tumor microenvironment in multiple myeloma
Source: Sci Rep. 2024 Jan 27;14:2264. doi: 10.1038/s41598-024-52922-7 (PMC10817968; doi:10.1038/s41598-024-52922-7)
Supplement: Supplementary file 15 — Supplementary Information 15. [file 41598_2024_52922_MOESM15_ESM.pdf]

1 AES
2 ANKRD28
3 ARL4C
4 ARPC1B
5 ATF7IP2
6 C1orf56
7 CCND1
8 CCND2
9 CFP
10 CRIP1
11 EEF1A1
12 ERLEC1
13 ERN1
14 FCGRT
15 GAPDH
16 GNLY
17 HIST1H1C
18 ISG15
19 MEI1
20 MGAT4A
21 NPM1
22 RHOH
23 RNF125
24 RPL14
25 RPLP0
26 S100A6
27 SPCS2
28 WARS
