# Supplementary material for: A neutrophil extracellular trap-related risk score predicts prognosis and characterizes the tumor microenvironment in multiple myeloma
Source: Sci Rep. 2024 Jan 27;14:2264. doi: 10.1038/s41598-024-52922-7 (PMC10817968; doi:10.1038/s41598-024-52922-7)
Supplement: Supplementary file 16 — Supplementary Information 16. [file 41598_2024_52922_MOESM16_ESM.pdf]

ANKRD28	-0.04657
ATF7IP2	-0.27249
C1orf56	0.102152
CCND1	-0.01497
CRIP1	0.116918
GAPDH	0.025477
HIST1H1C	0.107486
MEI1	-0.22581
MGAT4A	-0.24394
NPM1	0.218997
RHOH	-0.03016
RNF125	0.570506
S100A6	0.0707
