# Supplementary material for: A neutrophil extracellular trap-related risk score predicts prognosis and characterizes the tumor microenvironment in multiple myeloma
Source: Sci Rep. 2024 Jan 27;14:2264. doi: 10.1038/s41598-024-52922-7 (PMC10817968; doi:10.1038/s41598-024-52922-7)
Supplement: Supplementary file 20 — Supplementary Information 20. [file 41598_2024_52922_MOESM20_ESM.pdf]

PMID:35688556
ALPL
BST1
CD93
CEACAM3
CREB5
CRISPLD2
CSF3R
CYP4F3
DYSF
FCAR
FCGR3B
CPPED1
FPR1
FPR2
G0S2
HIST1H2BC
HPSE
CXCR1
CXCR2
KCNJ15
LILRB2
MGAM
MME
PDE4B
S100A12
SIGLEC5
SLC22A4
SLC25A37
TECPR2
TNFRSF10C
VNN3
AKT1
AKT2
ATG7
CLEC6A
CSF3
CTSG
CYBB
DNASE1
ELANE
ENTPD4
F3
HMGB1
IL17A
IL1B
IL6
IL8
IRAK4
ITGAM
ITGB2
KCNN3
MAPK1
MAPK3
MMP9
MPO
MTOR

PADI4
PTAFR
PIK3CA
RIPK1
RIPK3
SELP
SELPLG
SIGLEC14
TLR2
TLR4
TLR7
TLR8
TNF
