# Supplementary material for: A neutrophil extracellular trap-related risk score predicts prognosis and characterizes the tumor microenvironment in multiple myeloma
Source: Sci Rep. 2024 Jan 27;14:2264. doi: 10.1038/s41598-024-52922-7 (PMC10817968; doi:10.1038/s41598-024-52922-7)
Supplement: Supplementary file 22 — Supplementary Information 22. [file 41598_2024_52922_MOESM22_ESM.pdf]

Activated CD8 T cell	NA	ADRM1	AHSA1	C1GALT1C1	CCT6B	CD37	CD3D	CD3E	CD3G	CD69	CD8A	CETN3	CSE1L	GEMIN6	GNLY	GPT2	GZMA	GZMH	GZMK	IL2RB	LCK	MPZL1	NKG7	PIK3IP1	PTRH2	TIMM13	ZAP70
Central memory CD8 T cell	NA	ACTN4	ADAM12	ADCY9	F13A1	FCER1G	FCGR3B	FGF7	FKBP4	GLUD1	GM2A	GUSB	IL1RN	NOL11	NTRK1	RARA	RNF128	SIGLEC1	TNFRSF11A	TOX4	UBA52	ULBP1
Effector memeory CD8 T cell	NA	ACAP1	APOL3	ARHGAP10	ATP10D	C3AR1	CCR5	CD160	CD55	CFLAR	CMKLR1	DAPP1	FCRL6	FLT3LG	GZMM	HAPLN3	HLA-DMB	HLA-DPA1	HLA-DPB1	IFI16	LIME1	LTK	NFKBIA	SETD7	SIK1	TRIB2
Activated CD4 T cell	NA	AIM2	BIRC3	BRIP1	CCL20	CCL4	CCL5	CCNB1	CCR7	DUSP2	ESCO2	ETS1	EXO1	EXOC6	IARS	ITK	KIF11	KNTC1	NUF2	PRC1	PSAT1	RGS1	RTKN2	SAMSN1	SELL	TRAT1
Central memory CD4 T cell	NA	ABHD3	AHNAK	ANXA2P2	AQP3	ATHL1	BMI1	BZW2	CD63	COL4A1	CYLD	ELMO2	FYN	GLIPR1	GSS	IFITM2	ITGB1	ITGB2	KLF5	LSP1	NDUFB9	PKM2	SFXN3	SIRPG	SMAD4	STX4	TRADD	VIM	XRCC6
Effector memeory CD4 T cell	NA	ATM	CASP3	CASQ1	CD300E	DARS	DOCK9	EXOSC9	EZH2	GDE1	IL34	NCOA4	NEFL	PDGFRL	PTGS1	REPS1	SCG2	SDPR	SIGLEC14	SIGLEC6	TAL1	TFEC	TIPIN	TPK1	UQCRB	USP9Y	WIPF1	ZCRB1
T follicular helper cell	NA	B3GAT1	CDK5R1	PDCD1	BCL6	CD200	CD83	CD84	FGF2	GPR18	CEBPA	CECR1	CLEC10A	CLEC4A	CSF1R	CTSS	DMN	DPP4	LRRC32	MC5R	MICA	NCAM1	NCR2	NRP1	PDCD1LG2	PDCD6	PRDX1	RAE1	RAET1E	SIGLEC7	SIGLEC9	TYRO3	CHST12	CLIC3	IVNS1ABP	KIR2DL2	LGMN
Gamma delta T cell	NA	ACP5	AQP9	BTN3A2	C1orf54	CARD8	CCL18	CD209	CD33	CD36	CDK5	IL10RB	KLRF1	LGALS1	MAPK7	KLHL7	KRT80	LAMC1	LCORL	LMNB1	MEIS3P1	MPL	FABP1	FABP5	FADD	MFAP3L	MINPP1	RPS24	RPS7	RPS9	DBNL	CCL13
Type 1 T helper cell	NA	CD70	TBX21	ADAM8	AHCYL2	ALCAM	B3GALNT1	BBS12	BST1	CD151	CD47	CD48	CD52	CD53	CD59	CD6	CD68	CD7	CD96	CFHR3	CHRM3	CLEC7A	COL23A1	COL4A4	COL5A3	DAB1	DLEU7	DOC2B	EMP1	F12	FURIN	GAB3	GATM	GFPT2	GPR25	GREM2	HAVCR1	HSD11B1	HUNK	IGF2	RCSD1	RYR1	SAV1	SELE	SELP	SH3KBP1	SIT1	SLC35B3	SIGLEC10	SKAP1	THUMPD2	TIGIT	ZEB2	ENC1	FAM134B	FBXO30	FCGR2C	STAC	LTC4S	MAN1B1	MDH1	MMD	RGS16	IL12A	P2RX5	CD97	ITGB4	ICAM3	METRNL	TNFRSF1A	IRF1	HTR2B	CALD1	MOCOS	TRAF3IP2	TLR8	TRAF1	DUSP14
Type 17 T helper cell	NA	IL17A	IL17RA	C2CD4A	C2CD4B	CA2	CCDC65	CEACAM3	IL17C	IL17F	IL17RC	IL17RE	IL23A	ILDR1	LONRF3	SH2D6	TNIP2	ABCA1	ABCB1	ADAMTS12	ANK1	ANKRD22	B3GALT2	CAMTA1	CCR9	CD40	GPR44	IFT80
Type 2 T helper cell	NA	ASB2	CSRP2	DAPK1	DLC1	DNAJC12	DUSP6	GNAI1	LAMP3	NRP2	OSBPL1A	PDE4B	PHLDA1	PLA2G4A	RAB27B	RBMS3	RNF125	TMPRSS3	GATA3	BIRC5	CDC25C	CDC7	CENPF	CXCR6	DHFR	EVI5	GSTA4	HELLS	IL26	LAIR2
Regulatory T cell	NA	CCL3L1	CD72	CLEC5A	FOXP3	ITGA4	L1CAM	LIPA	LRP1	LRRC42	MARCO	MMP12	MNDA	MRC1	MS4A6A	PELO	PLEK	PRSS23	PTGIR	ST8SIA4	STAB1
Activated B cell	NA	ADAM28	CD180	CD79B	BLK	CD19	MS4A1	TNFRSF17	IGHM	GNG7	MICAL3	SPIB	HLA-DOB	IGKC	PNOC	FCRL2	BACH2	CR2	TCL1A	AKNA	ARHGAP25	CCL21	CD27	CD38	CLEC17A	CLEC9A	CLECL1
Immature B cell	NA	CD22	CYBB	FAM129C	FCRL1	FCRL3	FCRL5	FCRLA	HDAC9	HLA-DQA1	HVCN1	KIAA0226	NCF1	NCF1B	P2RY10	SP100	TXNIP	STAP1	TAGAP	ZCCHC2
Memory B cell	NA	AICDA	CCNA2	CDKN3	CLCN5	ENPP1	FCER1A	FCRL4	MYC	RUNX2	SORL1	SOX5	STAT5A	STAT5B	TLR9
Natural killer cell	NA	AKT3	AXL	BST2	CDH2	CRTAM	CSF2RA	CTSZ	CXCL1	CYTH1	DAXX	DGKH	DLL4	DPYD	ERBB3	F11R	FAM27A	FAM49A	FASLG	FCGR1A	FN1	FSTL1	FUCA1	GBP3	GLS2	GRB2	LST1	BCL2	CDC5L	FGF18	FUT5	FZR1	GAGE2	IGFBP5	KANK2	LDB3
CD56bright natural killer cell	NA	ABAT	C11orf75	C5orf15	CDHR1	DCAF12	DYNLL1	GPR137B	HCP5	HDGFRP2	KRT86	MLST8	ELMOD3	ENTPD5	FAM119A	FAM179A	CLIC2	COX7A2L	CREB3L4	CSF1	CSNK2A2	CSTA	CSTB	CTPS	CTSD	FST	GATA2	GMPR	HDC	HEY1	HOXA1	HS2ST1	HS3ST1	BCL11B	CDH3	MYL6B	NAA16	ClQA	ClQB	CYP27B1	EIF3M

CD56dim natural killer cell NA CYP27A1 DDX55 DYRK2 RPL37A NOTCH3 AKR7A3 GPRC5C GRIN1 HLA-E PORCN PSMC4 UPP1 IL21R KIR2DS1 KIR2DS2 KIR2DS5
Myeloid derived suppressor cell NA CCR2 CD14 CD2 CD86 CXCR4 FCGR2A FCGR2B FCGR3A FERMT3 GPSM3 IL18BP IL4R ITGAL ITGAM PARVG PSAP PTGER2 PTGES2 S100A8 S100A9
Natural killer T cell NA BTN2A2 CD101 CD109 CNPY3 CNPY4 CREB1 CRTC2 CRTC3 CSF2 KLRC1 FUT4 ICAM2 IL32 LAMP2 LILRB5 KLRG1 HSPA4 HSPB6 ISM2 ITIH2 KDM4C KIR2DS4 KIRREL3 SDCBP NFATC2IP MICB KIR2DL1 KIR2DL3 KIR3DL1 KIR3DL2 NCR1 FOSL1 TSLP SLC7A7 SPP1 TREM2 UBASH3A YBX2 CCDC88A CLEC1A THBD PDPN VCAM1 EMR1
Activated dendritic cell NA ABCD1 C1QC CAPG CCL3L3 CD207 CD302 ATP5B ATP5L ATP6V1A BCL2L1 C1QB SNURF SPCS3 CCNA1 CEACAM8 NOS2 SRA1 TNFRSF6B TREM1 TREML1 RHOA SLC25A37 TNFSF14 TREML4 VNN2 XPO6 CLEC4C TNFAIP2 UBD ACTR3 RAB1A SLA HLA-DQA2 SIGLEC5 SLAMF9
Plasmacytoid dendritic cell NA CBX6 DAB2 DDX17 HIGD1A IDH3A IL3RA MAGED1 NUCB2 OFD1 OGT PDIA4 SERTAD2 SIRPA TMED2 ENG FCAR IGF1 ITGA2B GABARAP GPX1 KRT23 PROK2 RALB RETNLB RNF141 SEC14L1 SEPX1 EMP3 CD300LF ABTB1 KLHL21 PHRF1
Immature dendritic cell NA ACADM AHCYL1 ALDH1A2 ALDH3A2 ALDH9A1 ALOX15 AMT ARL1 ATIC ATP5A1 CAPZA1 LILRA5 RDX RRAGD TACSTD2 INPP5F RAB38 PLAU CSF3R SLC18A2 AMPD2 CLTB C1orf162
Macrophage NA AIF1 CCL1 CCL14 CCL23 CCL26 CD300LB CNR1 CNR2 EIF1 EIF4A1 FPR1 FPR2 FRAT2 GPR27 GPR77 RNASE2 MS4A2 BASP1 IGSF6 HK3 VNN1 FES NPL FZD2 FAM198B HNMT SLC15A3 CD4 TXNDC3 FRMD4A CRYBB1 HRH1 WNT5B
Eosinophil NA GIPR KRT18P50 LRMP FOSB RRP12 GPR183 NR4A3 ST3GAL6 DEPDC5 PDE6C PKD2L2 GPR65 IL5RA P2RY14 DACH1 DAPK2 EMR3
Mast cell NA ADAMTS3 CPA3 CMA1 CTSG ARHGAP15 CPM FCN1 FTL HSPA6 ITGA9 RNASE3 S100A4 SIGLEC8 SLC6A4 PTGS2 EGR3 PILRA
Monocyte NA ASGR2 CFP ASGR1 CD1D UPK3A ACTG1 ANXA5 ATP6V1B2 CFL1 DAZAP2 CTBS EMR4P HIVEP2 MARCKSL1 MBP MMP15 PNPLA6 TMBIM6 PQBP1 TEX264 IKZF1
Neutrophil NA CREB5 CDA CHST15 S100A12 APOBEC3A CASP5 MMP25 HAL C1orf183 FFAR2 MAK CXCR1 STEAP4 MGAM BTNL8 CXCR2 TNFRSF10C VNN3
