# Supplementary material for: A neutrophil extracellular trap-related risk score predicts prognosis and characterizes the tumor microenvironment in multiple myeloma
Source: Sci Rep. 2024 Jan 27;14:2264. doi: 10.1038/s41598-024-52922-7 (PMC10817968; doi:10.1038/s41598-024-52922-7)

Experimental materials

1. Laboratory equipment

Name	Factory	Model number
Micropipettes	Eppendorf, Germany	/
Carbon dioxide incubator	Thermo	3111
Biological safety cabinet	SuJingAnTai	BS-1300IIA2
Inverted fluorescence microscope	OLYMPUS	CKX41
Centrifuges	Thermo	MICROCL 17
Real-time fluorescence quantitative PCR instrument	illumina eco	eco
Fluorescent PCR Tubes	EXTRAGENE/ illumina	/
Enzyme marker	Biotek	
Electrothermal constant temperature water bath	Beijing Changfeng Instrumentation Co.	HW-SY11-K P2
Aquapro Super Pure Water Meter	Aquapro	AJY-0501
PCR instrument	Dongsheng Innovative Biotechnology Co.	EDC-810
1ml RNase-free bagged tips	EXTRAGENE	/
200ul RNase-free bagged tips	EXTRAGENE	
10ul RNase-free bagged tips	EXTRAGENE	

2. Main reagents

Name	Factory	Item number
TRIzol	Invitrogen	15596-018
Isopropanol	Sangon Biotech	A507048
Trichloromethane	SINOPHARM	100006818
Anhydrous ethanol	Sangon Biotech	A500737
DEPC treated water	Sangon Biotech	B501005
cDNA first strand synthesis kit	TransGen Biotech	AT341
AceQTM Universal SYBR qPCR Master Mix	Vazyme	Q511-03

3. Primer sequences

Primer name	Primer sequence (5'→3')	Amplification product (bp)
H-GAPDH-F	GGAGCGAGATCCCTCCAAAAT	197
H-GAPDH-R	GGCTGTTGTCATACTTCTCATGG	
H-C1orf56-F	CAAGCCTTGCACCTATCAACA	81
H-C1orf56-R	GTGTCAGTACAGAGACTTGTGTC	
H-CRIP1-F	CCTGCCTGAAGTGCGAGAAAT	115
H-CRIP1-R	CCTTTAGGCCCAAACATGGC	
H-HIST1H1C-F	CCGCCTCTAAAGAGCGTAGC	106
H-HIST1H1C-R	AGACCAAGTTTGATACGGCTG	

H-RNF125-F	TGCCGTTTAATACCCGATGAGA	79
H-RNF125-R	ACAAAGTGTGACTAACTTGCAGA	
H-S100A6-F	GGGAGGGTGACAAGCACAC	79
H-S100A6-R	AGCTTCGAGCCAATGGTGAG	

Experimental procedure

1. Cell culture and cell passaging

1) Cells were observed under an inverted microscope to assess the extent of cell confluence as well as to confirm the absence of bacterial and fungal contamination;
2) Aspirate the culture solution from the petri dish (bottle);
3) Add PBS equal to half the volume of the culture solution to wash the monolayer of cells, usually three times;
4) Add 0.25% trypsin at 1mL/25cm^2 surface area to digest monolayer cells. Shake the petri dish (bottle) so that trypsin covers the monolayer of cells adequately;
5) Place the petri dish (bottle) back into the incubator and leave for 2-10 min;
6) The cells were observed with an inverted microscope, and when gaps appeared between the cells and the cells were not yet detached, the medium was gently aspirated to allow the trypsin remaining on the cell surface to further dissociate the cells sufficiently;
7) Disperse the cells by blowing with a small amount of culture medium containing fresh serum and remove 100-200 μl for counting;
8) Transfer the desired number of cells to a newly labeled petri dish (bottle) containing pre-warmed complete medium; select appropriate culture conditions to culture the cell line;
9) Repeat this step according to the growth characteristics of the cell line until the cells are in good condition, free of contamination and ready to be used in spread plates, with the rest selected for freezing.

2. Live cell count

1) A vial of passaged cells was taken and trypsin digested to prepare a cell suspension.
2) Tapan Blue Staining: Pipette appropriate amount of cell suspension into a centrifuge tube, add equal volume of Tapan Blue Staining Solution, gently blow to mix, dead cells will be

stained blue, while the viable cells refuse to be stained, and the number of refused cells will be counted within 10 minutes (the number of dead cells will be increased if the number of cells is exceeded for more than 10 minutes). If the counting is purely for cell counting without considering the viability of the cells, Tapan Blue Staining may not be used.

3) Take a set of blood counting plates, cover the coverslip, and blow the cell suspension through the side of the coverslip so that the cell suspension just fills the counting platform, taking care that there are no air bubbles under the coverslip and that you don't blow too much of the suspension and have it flow into the side slots.
4) Count the number of cells in the four large compartments: Place the blood counting plate under low magnification of the microscope and move the plate, when you see the counting squares appear in the mirror, count the number of cells in the large compartments in the four corners (each containing 16 medium compartments) that are not colored by the stain. Note that when counting, "count up, not down, count left, not right" for cells with pressed edges.
5) Calculate the number of cells in the original cell suspension. Calculate the cell density according to the formula below:

(number of cells in cell suspension)/mL = (number of cells in four large grids/4) $\times 2 \times 10^4$

The multiplication by 2 is due to the fact that 1:1 staining was performed using Tapan Blue, which is equivalent to a 2-fold dilution of the original cell suspension. If a dilution was performed in addition to the Tapan Blue staining, the corresponding dilution should be multiplied when calculating the cell density.

3. Cell processing

Treatments were performed according to the experimental plan.

**Experimental Grouping**	**1**	**2**	**3**
HS-5 Human Bone Marrow Stromal Cells	+	-	-
MM.1R Human Multiple Myeloma Cells	-	+	-
MM.1S Human Multiple Myeloma Cells	-	-	+

4. RNA extraction

1) 30mm cell culture dish for cell growth to 80% confluence, aspirate the culture supernatant, add 1mL TRIzol, shake the dish to make TRIzol fully cover the cells, room temperature static lysis for

10min, use the enzyme-free and sterile tip of the gun to blow fully and then transfer the cell lysate to an enzyme-free and sterile 1.5mL EP tube;

2) Add 200 μL of chloroform per 1 mL of cell lysate, shake the EP tube vigorously up and down by hand for 30 s, and let it stand at room temperature for 2-3 min;

3) Centrifugation at 4°C, 12000g x 15min-20min;

4) Transfer the upper aqueous phase (about 400 μL) to a new enzyme-free and sterile 1.5 mL EP tube, add 600 μL of isopropanol, mix upside down and let stand for 10 min at room temperature;

5) Centrifuge at 4°C, 12,000g x 10min;

6) Discard the supernatant, and the precipitate was washed three times with 1 mL of pre-cooled 70% anhydrous ethanol, air-dried for 5-10 min, and dissolved in 20 μL DEPC treated water;

7) The RNA solution was diluted 20-fold, and the RNA concentration was detected and calculated spectrophotometrically.

Batch 1 samples

**Experimental Grouping**	**260 Raw**	**280 Raw**	**320 Raw**	**260**	**280**	**260/280**	**ng/μL**
1	0.517	0.318	0.071	0.472	0.262	1.805	377.787
2	0.684	0.424	0.102	0.577	0.319	1.808	461.24
3	0.883	0.502	0.081	0.796	0.419	1.901	636.92
4	0.6	0.349	0.062	0.565	0.3	1.881	452.06
5	0.616	0.358	0.068	0.579	0.306	1.89	463.43
6	0.828	0.487	0.086	0.783	0.423	1.852	626.67
7	0.768	0.457	0.09	0.721	0.389	1.854	576.71
8	0.659	0.394	0.084	0.605	0.325	1.861	484.23

5. Reverse transcription

1) On ice, nuclease-free PCR tubes were filled with the following reagents:

total RNA (1μg)	X μL
5×TransScript All-in-One SuperMix for qPCR	4 μL
gDNA Remover	1 μL
RNAse-free ddH_2O	to 20 μL

2) Mix gently and incubate at 42°C for 15 min.

3) Inactivate TransScript RT/RI and gDNA Remover by heating at 85°C for 5s.

4) Store the above solution at -20°C.

6. Fluorescent quantitative PCR assay

1) A 10-fold dilution of the cDNA sample was used as a template for on-line assay.

2) Preparation of reaction mixtures

**Reaction Component**	**Concentration**	**Volume(μL)**
SybrGreen qPCR Master Mix	2×	10
Primer F (10μM)	10μM	0.4
Primer R (10μM)	10μM	0.4
ddH_2O		7.2
Template (cDNA)		2
Total 20μL		

3) PCR cycle program settings

Stage 1	Premutability	Reps: 1	95°	5 min
Stage 2	Cyclic response	Reps: 40	95°<br>60°	10 sec<br>30 sec
Stage 3	Melting Curve	Reps: 1	95°<br>60°<br>95°	15 sec<br>60 sec<br>15 sec

4) Operation of the instrument

After completing the above steps, the sample-spiked 96-well plate was placed in an ABI quantstudio5 fluorescent quantitative PCR instrument for reaction.

Statistical analysis

If three independent replications were performed in this experiment, statistical analyses can be performed using one-way ANOVA for unpaired samples with the following statistical paths:

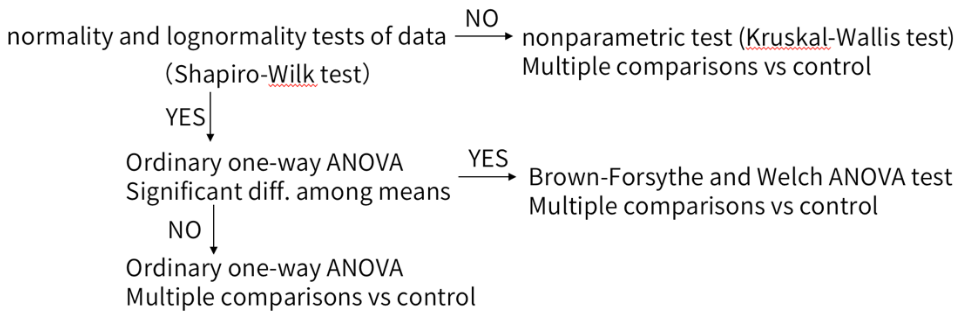

Supplement: Supplementary file 23 — Supplementary Information 23. [file 41598_2024_52922_MOESM23_ESM.pdf]
